# Directed Supramolecular Organization of N-BAR Proteins through Regulation of H0 Membrane Immersion Depth

**DOI:** 10.1038/s41598-018-34273-2

**Published:** 2018-11-06

**Authors:** Osman Kahraman, Ralf Langen, Christoph A. Haselwandter

**Affiliations:** 1 Department of Physics & Astronomy and Molecular and Computational Biology Program, Department of Biological Sciences, University of Southern California, Los Angeles, CA 90089 USA; 2 Department of Biochemistry and Molecular Biology, Zilkha Neurogenetic Institute, Keck School of Medicine, University of Southern California, Los Angeles, CA 90033 USA; 3 Present Address: R&D Center, Arcelik A.S., Tuzla, Istanbul, 34950 Turkey

## Abstract

Many membrane remodeling events rely on the ability of curvature-generating N-BAR membrane proteins to organize into distinctive supramolecular configurations. Experiments have revealed a conformational switch in N-BAR proteins resulting in vesicular or tubular membrane shapes, with shallow membrane immersion of the H0 amphipathic helices of N-BAR proteins on vesicles but deep H0 immersion on tubes. We develop here a minimal elastic model of the local thinning of the lipid bilayer resulting from H0 immersion. Our model predicts that the observed conformational switch in N-BAR proteins produces a corresponding switch in the bilayer-mediated N-BAR interactions due to the H0 helices. In agreement with experiments, we find that bilayer-mediated H0 interactions oppose N-BAR multimerization for the shallow H0 membrane immersion depths measured on vesicles, but promote self-assembly of supramolecular N-BAR chains for the increased H0 membrane immersion depths measured on tubes. Finally, we consider the possibility that bilayer-mediated H0 interactions might contribute to the concerted structural reorganization of N-BAR proteins suggested by experiments. Our results indicate that the membrane immersion depth of amphipathic protein helices may provide a general molecular control parameter for membrane organization.

## Introduction

**Figure 1:**
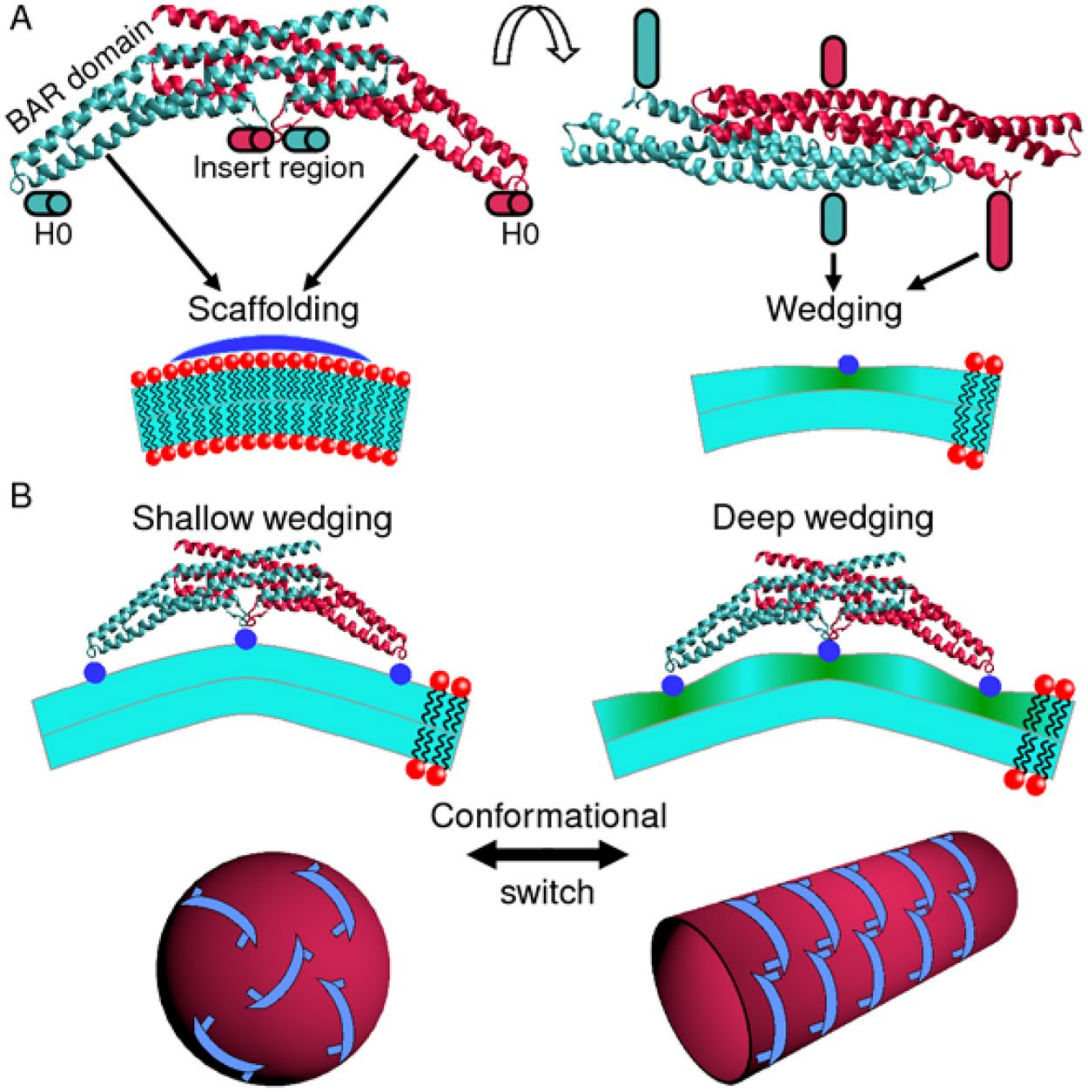
Illustration of membrane curvature generation by N-BAR proteins. (**A**) Molecular structure of the N-BAR protein endophilin (PDB ID code 2C08) viewed perpendicular (upper left panel) and parallel (upper right panel) to the plane of the membrane. Endophilin employs the scaffolding (lower left panel) and wedging (lower right panel) mechanisms for membrane curvature generation through its BAR domain and through its N-terminal H0 helices and insert region, respectively. The molecular structure of amphiphysin is highly homologous to that of endophilin but lacks the insert region^10,11,15^. (**B**) A conformational switch controls generation of isotropic (vesicular) and anisotropic (tubular) membrane curvatures by N-BAR proteins^22–24^, with shallow wedging and little scaffolding on vesicles (left panel) but deep wedging and pronounced scaffolding on tubes (right panel). In the membrane cross sections, green shading indicates membrane regions in which deep membrane immersion of amphipathic helices^22,23^ induces compression of the lipid bilayer hydrophobic thickness^42–50^.

The remodeling of cellular membrane shape is often regulated^1–5^ by curvature-generating membrane proteins adsorbing onto one lipid bilayer leaflet. Biologically important examples of membrane remodeling are provided by synaptic endocytosis and T-tubule formation^5–9^, where N-BAR proteins such as amphiphysin and endophilin deform the cell membrane to generate highly curved cylindrical tubes and spherical vesicles. The ability of N-BAR proteins to induce tubular and spherical membrane shapes has been confirmed through *in vitro* experiments, which have shown^10–15^ that amphiphysin and endophilin can generate highly curved lipid bilayer tubes and vesicles with diameter ≈15–50 nm from large vesicles with diameter >100 nm. N-BAR proteins are homo- or heterodimers with a highly homologous structure consisting of the crescent-shaped BIN/amphiphysin/Rvs (BAR) domain and the N-terminal amphipathic H0 helices located close to the tips of the BAR domain^10,11,15–18^ (see Fig. 1A). The structure of N-BAR proteins^10,11,15–18^ suggests two key molecular mechanisms^1–5,19–21^ for generation of membrane curvature by N-BAR proteins: scaffolding and wedging (Fig. 1A). In the scaffolding mechanism, curved proteins deform the membrane by binding to one of the two lipid bilayer leaflets. In the wedging mechanism, proteins partially insert amphipathic helices into one lipid bilayer leaflet, making it favorable for the lipid bilayer to be curved. N-BAR proteins induce^1–5,10,11,15–18^ scaffolding via their BAR domains and wedging via their H0 helices (Fig. 1A). Some N-BAR proteins such as endophilin, but not amphiphysin, also interact with the membrane through wedging of additional amphipathic helices (Fig. 1A).

A remarkable feature of membrane remodeling by N-BAR proteins is that a single protein species can induce distinct types of membrane curvature, such as the isotropic and anisotropic curvatures associated with lipid bilayer vesicles and tubes, respectively. Electron paramagnetic resonance (EPR) experiments^22–24^ have revealed that a conformational switch in the molecular structure of the membrane-bound protein controls the competition between generation of highly curved isotropic (vesicular) and anisotropic (tubular) membrane shapes by amphiphysin and endophilin (see Fig. 1B). In particular, vesicle-bound N-BAR proteins interact with the lipid bilayer through shallow wedging of the H0 helices with little or no scaffolding (left panel of Fig. 1B), while tube-bound N-BAR proteins show a combination of scaffolding and increased wedging depths of the H0 helices (right panel of Fig. 1B). However, it is unknown how the observed conformational switch in N-BAR proteins^22–24^ generates the observed isotropic and anisotropic membrane curvatures^1–5,10,11,15,25^. A key challenge is that membrane remodeling by N-BAR proteins is a collective phenomenon requiring a threshold N-BAR concentration and distinctive supramolecular arrangements of N-BAR proteins^5,10–14,22–24,26–33^, and therefore results from many interacting proteins rather than individual, isolated proteins. In particular, generation of vesicles by N-BAR proteins is thought to rely on membrane-bound N-BAR proteins being oriented in a range of different directions (left panel of Fig. 1B) while, on tubes, N-BAR proteins form supramolecular, locally ordered tip-to-tail chains with antiparallel alignment and dimerization of the H0 helices of neighboring N-BAR proteins^5,12,22–24,26–28,31–33^ (right panel of Fig. 1B).

How does the observed conformational switch in N-BAR proteins control the supramolecular organization of N-BAR proteins? Experiments have suggested^5,26,27,33^ that nonspecific interactions between the H0 helices of neighboring N-BAR proteins are essential for the self-assembly, local ordering, and stability of supramolecular chains of N-BAR proteins. Indeed, BAR proteins lacking the H0 helices are also able to generate tubes, but show supramolecular configurations distinct from those of N-BAR proteins^2,5^. In the case of integral membrane proteins spanning the lipid bilayer, bilayer-thickness-mediated protein interactions originating from a bilayer-protein hydrophobic thickness mismatch have been found^34–41^ to provide a general physical mechanism underlying protein organization. A wide range of experiments^42–47^ and molecular dynamics (MD) simulations^42–44,48–50^ have shown that membrane immersion of amphipathic protein wedges can substantially decrease the lipid bilayer thickness. Unlike integral membrane proteins, however, amphipathic protein wedges only insert partially into one lipid bilayer leaflet (Fig. 1). We show here how the classic elastic model describing bilayer-thickness-mediated interactions between integral membrane proteins^34–41^ can be generalized to obtain a minimal elastic model of lipid bilayer-mediated wedge interactions. Our model provides a quantitative link between the observed structures of N-BAR proteins^10,11,15–18,22,23^ and the distinctive supramolecular arrangements of N-BAR proteins yielding isotropic and anisotropic membrane curvatures^5,12,22–24,26–28,31–33^. With the key model parameters estimated directly from experiments, our model predicts that the shallow H0 membrane immersion depths measured on vesicles^22,23^ result in bilayer-mediated H0 interactions between N-BAR proteins that oppose the self-assembly of N-BAR chains through H0 dimerization^5,26–28^, thus allowing a variety of different N-BAR orientations on vesicles (left panel of Fig. 1B). In contrast, our model predicts that the increased H0 membrane immersion depths measured on tubes^22,23^ result in bilayer-mediated H0 interactions between N-BAR proteins that promote the self-assembly of locally ordered tip-to-tail N-BAR chains^5,12,26–28,33^ via the observed antiparallel alignment and dimerization of the H0 helices of neighboring N-BAR proteins (right panel of Fig. 1B). Finally, we consider the possibility that bilayer-mediated H0 interactions might contribute to the concerted structural reorganization of N-BAR proteins suggested by experiments^13,14,22–24^, and may thus help to initiate membrane remodeling. Our results only rely on generic features of bilayer-wedge interactions and therefore suggest that, in analogy to the sorting of integral membrane proteins through bilayer-protein hydrophobic thickness mismatch^34–41^, the membrane immersion depth of amphipathic helices^1–5^ may provide a general molecular control parameter for the supramolecular organization of membrane proteins with amphipathic protein wedges.

## Elastic model of bilayer-mediated wedge interactions

**Figure 2:**
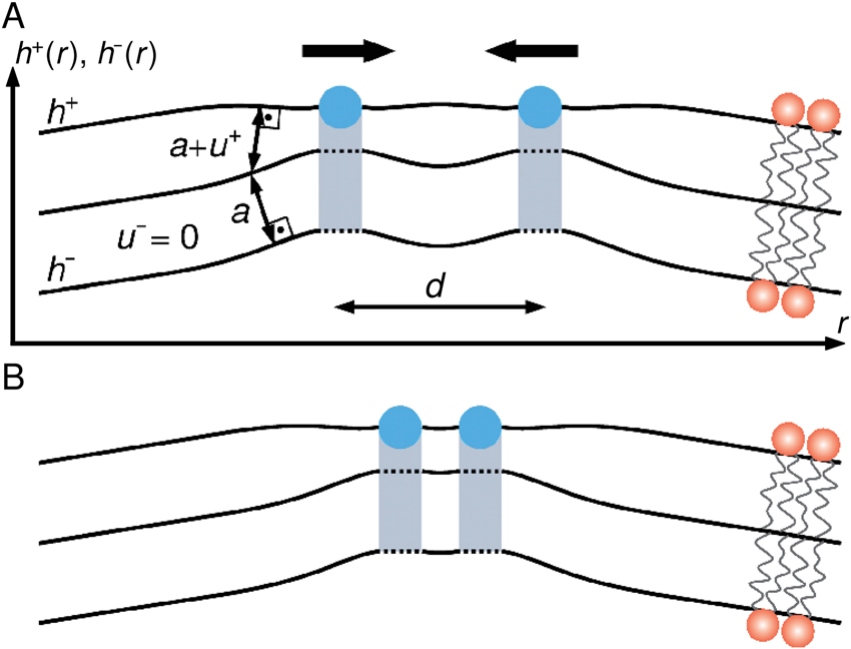
Schematic of bilayer-mediated interactions between protein wedges. (**A**) Amphipathic helices (viewed parallel to the helix axes and indicated in blue) deform the shape *h*^+^ and hydrophobic thickness *u*^+^ +*a* of the upper lipid bilayer leaflet, and may indirectly perturb *h*^−^ and *u*^−^ in the lower leaflet via the coupling between upper and lower leaflets. We set here *u*^−^ = 0 (see main text). For simplicity, we assume in this schematic that the H0 helices are in the face-on orientation, and that the H0-induced membrane deformations are only a function of the spatial coordinate *r* measured perpendicular to the helix axes. Equation (3) predicts that, for large enough helix immersion depths, two protein wedges can be attracted to each other by bilayer-mediated wedge interactions (black arrows). (**B**) Our elastic model of bilayer-mediated wedge interactions predicts that, for large enough helix immersion depths, the distance *d* separating the axes of two neighboring amphipathic helices can take an optimal value set by the key lipid and protein properties captured by equation (3).

We develop in this section a minimal elastic model of wedge-induced lipid bilayer thickness deformations in which, akin to the classic Helfrich-Canham-Evans model of bilayer-scaffold interactions^19–21,51–54^, the key model parameters can be directly estimated from experiments. Key structural parameters characterizing bilayer-wedge interactions are the wedge immersion depth and the wedge size^10,19,22,23,51,55–58^. Protein wedges only directly interact with one leaflet of the lipid bilayer, which we take to correspond to the upper leaflet, with the wedge-induced deformation of the other leaflet being determined by the coupling between upper and lower leaflets (Fig. 1). Contrary to the classic elastic model describing bilayer-protein interactions for integral membrane proteins^34–41^, protein wedges are expected to induce distinct, asymmetric deformations in the two bilayer leaflets. Following recent generalizations of the continuum elasticity theory of lipid bilayers^59–62^, we separately account for wedge-induced deformations in each bilayer leaflet through the fields *h*^±^ (*x*, *y*) and *u*^±^ (*x*, *y*) capturing the height and thickness deformations in the upper and lower lipid bilayer leaflets (see Fig. 2). Expansion of the leaflet elastic energies in terms of the lipid area deformation and the leaflet curvatures up to second order^61^ yields the following energy cost of wedge-induced lipid bilayer deformations (see Supplementary Information Sec. S1):

1 $$G=\frac{1}{2}\,\int {\rm{d}}x{\rm{d}}y\,\{\frac{{K}_{b}}{2}[{({\nabla }^{2}{h}^{+})}^{2}+{({\nabla }^{2}{h}^{-})}^{2}]+\frac{{K}_{t}}{2}[{(\frac{{u}^{+}}{a})}^{2}+{(\frac{{u}^{-}}{a})}^{2}]+\tau (\frac{{u}^{+}}{a}+\frac{{u}^{-}}{a})+\,\frac{\tau }{2}[{(\nabla {h}^{+})}^{2}+{(\nabla {h}^{-})}^{2}]\},$$

where *K*_*b*_ is the lipid bilayer bending rigidity, *K*_*t*_ is the lipid bilayer thickness deformation modulus, *a* is the thickness of the unperturbed monolayer hydrophobic core, and *τ* is the membrane tension. To ensure that no overlaps and voids occur within the lipid bilayer, the deformation fields in equation (1) are required to satisfy a bilayer continuity constraint^61^ (Fig. 2) which, to leading order, is given by

2 $${h}^{+}-{h}^{-}={u}^{+}+{u}^{-}+2a.$$

The effective parameters *K*_*b*_, *K*_*t*_, and *a* in equations (1) and (2) characterize the mechanical properties of lipid bilayers and can be directly estimated from, for instance, micropipette aspiration and x-ray diffraction experiments. Typical values of these parameters for phospholipid bilayers are *K*_*b*_ ≈ 20 *k*_*B*_*T*, *K*_*t*_ ≈ 60 *k*_*B*_*T/*nm^2^, and *a* ≈ 2.0 nm^36,63^, which we used for all the numerical results obtained here. The terms with coefficient *K*_*b*_ in equation (1) describe the energy cost of wedge-induced leaflet bending deformations, and generalize the standard elastic model of protein-induced bilayer bending deformations^34–36,40,64–66^ to scenarios in which the two bilayer leaflets do not necessarily show identical bending deformations. Similarly, the *K*_*t*_- and *τ*-terms in equation (1) generalize the standard elastic models of bilayer thickness deformations and membrane tension^34–36,40,64–66^ to allow for asymmetric deformations of the bilayer leaflets. In the special case of symmetric bilayer thickness deformations, *u*^+^ = *u*^−^, equation (1) with equation (2) reduces to the classic elastic model of bilayer-protein interactions for integral membrane proteins^34–41,64–66^.

A variety of experiments^42–47^ and MD simulations^42–44,48–50^ have shown that insertion of amphipathic protein wedges into the membrane can substantially decrease the lipid bilayer thickness. Following this previous work, we assume here that the H0 helices of N-BAR proteins can locally compress the bilayer leaflet into which they are inserted. For simplicity, we further assume that, in the vicinity of the H0 helices, the magnitude of the H0-induced leaflet compression approximately matches the membrane immersion depth of the H0 helices so as to optimize the electrostatic and hydrophobic interactions between the H0 helices and the membrane. In particular, previous studies^22,23,45,46^ indicate that, if a protein wedge is inserted into the membrane, the lipids surrounding the protein wedge tend to compress so that the negatively charged moieties of the lipid head groups are in contact with the hydrophilic, positively charged portion of the wedge surface, and the lipid tails shield the hydrophobic portion of the wedge surface. This suggests that the observed membrane immersion depths of the H0 helices of N-BAR proteins^22,23^, which are measured with respect to the unperturbed lipid bilayer thickness, take a similar magnitude as the compression of the lipids surrounding the H0 helices (Fig. 2). However, this assumption is not essential for our model of bilayer-mediated wedge interactions, and we study here the dependence of bilayer-mediated H0 interactions on the magnitude of H0-induced leaflet compression. This allows us to arrive at a general understanding of how H0-induced leaflet compression affects N-BAR organization—even if the assumption that the magnitude of H0-induced local leaflet compression matches the H0 membrane immersion depth is not entirely accurate—and, more generally, permits us to estimate the overall trends by which wedge-induced alterations in lipid bilayer thickness affect proximity between wedges in other protein systems. We note that the large membrane immersion depths of the H0 helices observed in experiments on N-BAR proteins may, at least in part, be stabilized by favorable interactions between the lipid bilayer and the BAR domains^22–24^.

The above picture of bilayer-wedge interactions suggests that, for large enough wedge immersion depths, an amphipathic protein wedge induces substantial local compression of the bilayer leaflet into which the protein wedge is inserted, with only minor thickness deformations in the opposite leaflet not directly interacting with the protein wedge^22,23,35,45,46^. We therefore simplify equation (1) by setting *u*^−^ = 0 which, together with equation (2), yields

3 $$G=\frac{1}{2}\int {\rm{d}}x{\rm{d}}y\{{K}_{b}\,[{({\nabla }^{2}{h}^{+})}^{2}-({\nabla }^{2}{h}^{+})({\nabla }^{2}{u}^{+})+\frac{1}{2}{({\nabla }^{2}{u}^{+})}^{2}]+\frac{{K}_{t}}{2}{(\frac{{u}^{+}}{a})}^{2}+\tau \frac{{u}^{+}}{a}+\tau [{(\nabla {h}^{+})}^{2}-(\nabla {h}^{+})\cdot (\nabla {u}^{+})+\frac{1}{2}{(\nabla {u}^{+})}^{2}]\}.$$

Note that the above elastic model of bilayer-wedge interactions involves, in addition to the bending, thickness deformation, and tension terms associated with *h*^+^ and *u*^+^ in equation (1)^34–36,40,64–66^, bending and tension terms penalizing leaflet deformations that induce opposite signs in the curvatures and gradients of *h*^+^ and *u*^+^, respectively. As a result, *h*^+^ and *u*^+^ are coupled to each other. Also, since we set *u*^−^ = 0 but allow for deformations of *h*^−^ (Fig. 2), the bending and tension terms associated with *u*^+^ in equation (3) are reduced by a factor of 1/2 with respect to the corresponding terms for *h*^+^, which effectively also involve contributions due to *h*^−^. Protein wedges may, in addition to the bilayer thickness deformations found previously^42–50^, also induce lipid tilt deformations. Our aim here is to explore the role of bilayer-mediated H0 interactions due to bilayer thickness deformations^42–50^ in N-BAR organization, and we therefore do not include lipid tilt deformations in equation (3).

**Figure 3:**
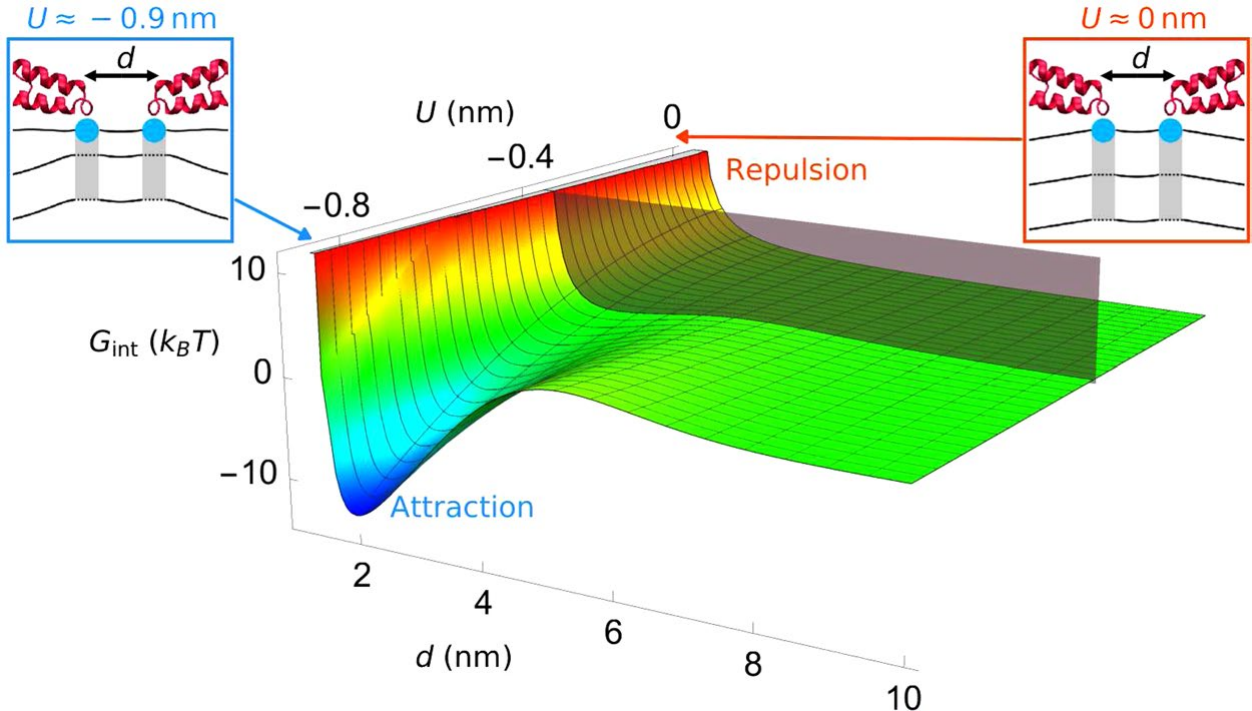
Bilayer-mediated interactions between H0 helices. Interaction potential *G*_int_ between two parallel H0 helices of neighboring N-BAR proteins versus the immersion depth of the two helices, *U*, and the distance separating the two H0 axes, *d*, for *τ* = 0. We obtained *G*_int_ from the exact analytic solution minimizing the energy cost of H0-induced lipid bilayer deformations in equation (3) (see the Methods section), which neglects boundary effects due to the H0 tips. *G*_int_ < 0 corresponds to favorable bilayer-mediated interactions between H0 helices, and *G*_int_ > 0 corresponds to unfavorable interactions. EPR experiments suggest *U* ≈ 0 nm and *U* ≈ −0.9 nm for vesicle- and tube-bound N-BAR proteins^22,23^ (see schematics in insets).

**Figure 4:**
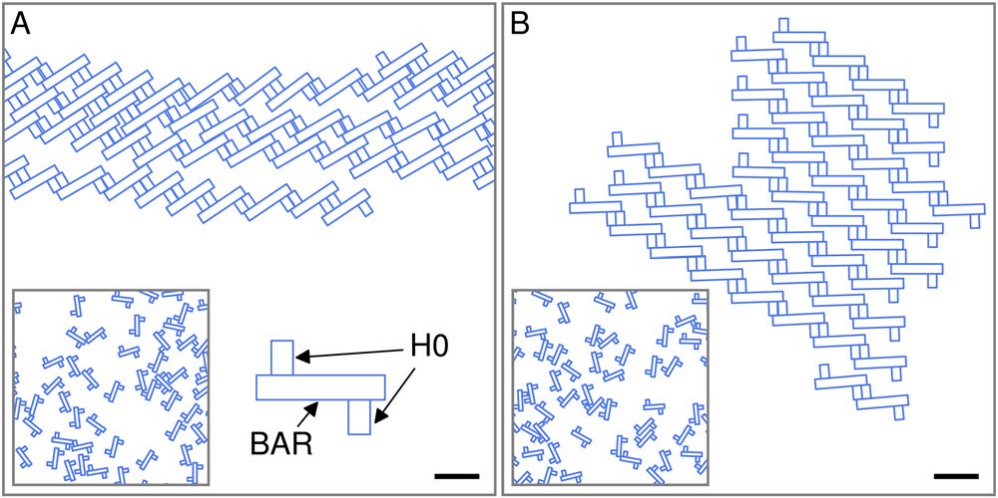
Multimerization of N-BAR proteins through bilayer-mediated interactions. Energetically favorable N-BAR arrangements obtained, for *U* ≈ −0.9 nm^22,23^ and *τ* = 0, from simulated annealing Monte Carlo simulations using (**A**) the N-BAR pair potential implied by the H0-induced leaflet thickness deformations in equation (3) with *h*^+^ = 0 and (**B**) a total N-BAR pair potential that is the sum of the potential used in (**A**) and a BAR pair potential based on previous calculations^53^ (see the Methods section). The left insets in (**A**) and (**B**) show the random initial N-BAR configurations employed for (**A**) and (**B**). We used the N-BAR shape shown in the right inset in (**A**), which we modeled after the N-BAR structures described previously^5^, with hardcore steric constraints (see Supplementary Information Sec. S6). We employed periodic boundary conditions, with the N-BAR proteins in (**A**) forming a single chain. Scale bars, 10 nm.

**Figure 5:**
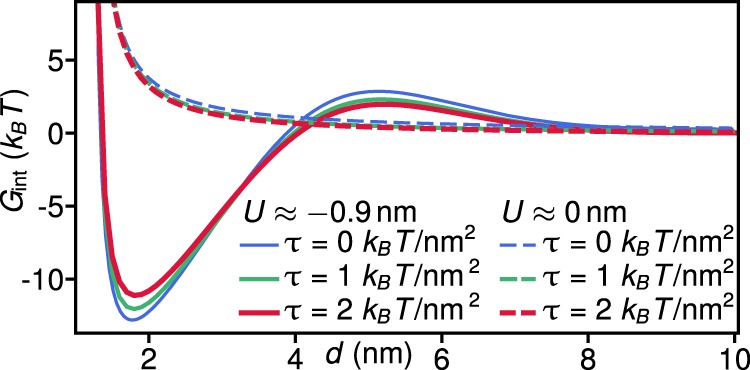
Effect of membrane tension on bilayer-mediated interactions between H0 helices. Interaction potential *G*_int_ between two parallel H0 helices of neighboring N-BAR proteins obtained from equation (3) as in Fig. 3 versus the distance separating the two H0 axes, *d*, for the deep (*U* ≈ −0.9 nm) and shallow (*U* ≈ 0 nm) immersion states of the H0 helices of N-BAR proteins^22,23^ at the indicated values of the membrane tension *τ*.

**Figure 6:**
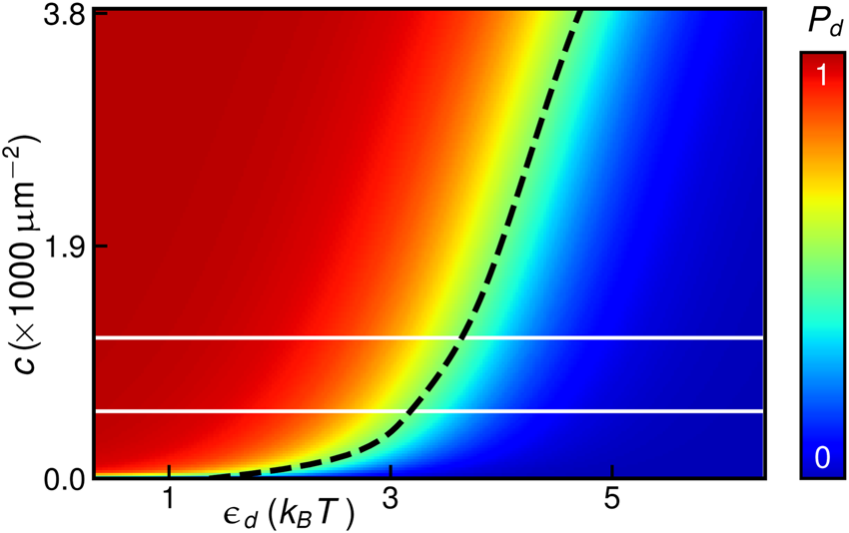
Concerted structural reorganization of N-BAR proteins through bilayer-mediated interactions between H0 helices. Probability of a pair of N-BAR proteins to be collectively in the observed conformational state with deep immersion of the H0 helices^22,23^, *P*_*d*_, obtained from equation (8) versus concentration of membrane-bound N-BAR proteins, *c*, and the energy difference between the observed N-BAR conformational states with deep and shallow immersion of the H0 helices^22,23^ in the absence of any interactions between N-BAR proteins, *ε*_*d*_. We analytically calculated the H0-induced N-BAR interactions following similar steps as for Fig. 3 (see the Methods section) and set *τ* = 0. The dashed black curve corresponds to *P*_*d*_ = 1/2 and, for reference, the solid white lines delineate the approximate range of N-BAR concentrations found to tubulate large vesicles *in vitro*^30^.

In analogy to bilayer-mediated interactions between integral membrane proteins^34–41,64–66^, overlapping wedge-induced lipid bilayer deformations are expected to give rise to bilayer-mediated interactions between protein wedges (Fig. 2). To calculate these interactions for Figs. 3–6, we proceed similarly as in the classic elastic model of bilayer-protein interactions for integral membrane proteins^34–41,64–66^ and minimize equation (3) with respect to *h*^+^ and *u*^+^ subject to suitable boundary conditions at the bilayer-wedge interface (see the Methods section). We use here the wedge length *L* ≈ 3 nm and the wedge radius *r*_0_ ≈ 0.6 nm associated with the H0 helices of endophilin^10,67^. As suggested by experiments and MD simulations^5,27^, we take the bilayer-H0 interactions to be uniform along the H0 helix axes. The most pronounced bilayer-mediated wedge interactions are expected to occur for wedges in the face-on orientation, for which the wedge interaction surface is maximized. Neglecting boundary effects due to the H0 tips, the energy density of bilayer-mediated wedge interactions can then be estimated from a one-dimensional representation of equation (3) with a single spatial coordinate *r* measured perpendicular to the helix axes (Fig. 2). We scale this energy density by *L* to obtain the total interaction energy (see the Methods section). With the exception of Fig. 4, where we test these assumptions and allow for arbitrary wedge orientations, we focus, throughout this Article, on wedges in the face-on orientation and neglect boundary effects due to the wedge tips. We have obtained, for wedges in the face-on orientation with negligible boundary effects, the exact analytic solution of the bilayer-mediated wedge interaction potential implied by equation (3) (see the Methods section), which we employ, with the exception of Fig. 4, throughout this Article. For Fig. 4, we resort to numerical methods to calculate the bilayer-mediated wedge interactions.

A key parameter in our model of bilayer-mediated wedge interactions is the wedge immersion depth *U*, where we set *u*^+^ = *U* at the bilayer-wedge interface. We thereby assume that the thickness of the lipid bilayer leaflet decreases in the vicinity of amphipathic protein wedges so as to match the wedge immersion depth. Based on EPR experiments on amphiphysin and endophilin^22,23^, we have the wedge immersion depths *U* ≈ 0 nm and *U* ≈ −0.9 nm for the shallow and deep immersion states of the H0 helices of N-BAR proteins. While, as discussed above, it is known that protein wedges can substantially decrease the lipid bilayer thickness^42–50^, the precise relation between helix immersion depth and local leaflet thickness deformations remains to be elucidated. Previous work on integral membrane proteins^68^ suggests that, for substantial protein-induced lipid bilayer thickness deformations, insertion of amphipathic helices may be alleviated by a combination of bilayer thickness deformations^42–50^ and (unfavorable) interactions between hydrophobic and hydrophilic surfaces. As a result, the value $|U|\approx 0.9$ nm estimated from EPR experiments for the deep H0 immersion state^22,23^ may be effectively reduced. We show, below, that the strength of favorable bilayer-mediated interactions between two H0 helices with membrane immersion depth *U* decreases with the value of $|U|$. However, we also find that there is a whole range of *U* yielding unfavorable and favorable bilayer-mediated H0 interactions, with unfavorable interactions for $|U| < |{U}_{c}|$ and favorable interactions for $|U| > |{U}_{c}|$, where *U*_*c*_ is the critical membrane immersion depth of the H0 helices governing the crossover from unfavorable to favorable bilayer-mediated H0 interactions. For the scenarios considered here we have *U*_*c*_ ≈ −0.4 nm at zero membrane tension, but the numerical value of *U*_*c*_ depends on the specific lipid and wedge properties under consideration (see Supplementary Information Table S1). We follow here previous theoretical and computational studies of protein-induced lipid bilayer deformations^31,32,34–36,40,53,54,64–66^ and assume that the unperturbed reference state of the lipid bilayer corresponds to a flat surface with *h*^+^ = *u*^+^ = 0, but have confirmed that similar results for H0-induced N-BAR interactions are obtained if the reference state of the bilayer is highly curved (see Supplementary Information Sec. S5).

## Results

### Helix immersion depth is crucial for bilayer-mediated H0 interactions

Equation (3) allows prediction of the contributions to bilayer-mediated N-BAR interactions due to the H0 helices. Other contributions to N-BAR interactions can arise, for instance, through bilayer-mediated interactions between the BAR domains^19–21,51–54^, which we return to in the next section. We focus, for now, on neighboring N-BAR proteins with H0 helices that face each other, which is expected to yield particularly pronounced bilayer-mediated H0 interactions, and neglect any boundary effects on the wedge-induced membrane deformations due to the H0 tips. We test these assumptions in the next section. The only relevant spatial dimension for H0-induced bilayer-mediated interactions between N-BAR proteins is then the distance *d* separating the axes of the neighboring H0 helices of the two N-BAR proteins, which we measure perpendicular to the H0 helix axes (Fig. 2). We have obtained the general analytic solution of the resulting H0-induced interaction potential between N-BAR proteins (see the Methods section), which is plotted in Fig. 3 for membranes with vanishing tension. With the key model parameters estimated directly from experiments, our model predicts that bilayer-mediated N-BAR interactions due to the H0 helices depend critically on the membrane immersion depth of the H0 helices. For shallow immersion depths *U* > −0.4 nm, we find that the H0 helices of neighboring N-BAR proteins repel each other. This can be understood by noting that, in this regime, the leaflet curvature deformations induced by the H0 helices dominate over leaflet thickness deformations, resulting in unfavorable bilayer-mediated protein interactions^36,40,52–54,65,66^. EPR experiments have shown^22,23^ that the H0 helices of vesicle-bound N-BAR proteins are at a similar height as the lipid phosphate headgroups in the unperturbed lipid bilayer, suggesting that *U* ≈ 0 nm. Our model therefore predicts that, on vesicles, bilayer-H0 interactions oppose the self-assembly of supramolecular N-BAR chains through H0 dimerization^5,26–28^, with an energy cost >10 *k*_*B*_*T* for each H0 dimer, thus allowing a variety of different N-BAR orientations on vesicles^22–24^.

For immersion depths *U* < −0.4 nm, in contrast, the interplay between protein-induced leaflet curvature and thickness deformations yields non-monotonic interactions between the H0 helices of neighboring N-BAR proteins. We find that, in this regime, H0-induced leaflet thickness deformations produce favorable lipid bilayer-mediated interactions between H0 helices at intermediate helix separations $1.5\,nm\,\lessapprox \,d\,\lessapprox \,5\,nm$, with unfavorable interactions at small and large helix separations, thus making it energetically favorable for H0 helices to reduce their bilayer deformation footprint by forming dimers. EPR measurements suggest that *U* ≈ −0.9 nm for tube-bound N-BAR proteins^22,23^, for which our model predicts that bilayer-H0 interactions strongly promote the self-assembly of supramolecular, locally ordered tip-to-tail N-BAR chains^5,12,26–28,33^ via the observed dimerization of the H0 helices of neighboring N-BAR proteins, with an energy gain >10 *k*_*B*_*T* for each H0 dimer. We find H0 dimerization energies >1 *k*_*B*_*T* for *U* < −0.5 nm. Figure 3 thus predicts that the observed conformational switch from shallow to deep wedging of the H0 helices^22,23^ yields a corresponding switch from unfavorable to favorable bilayer-mediated N-BAR interactions due to the H0 helices, thereby allowing a variety of different orientations of neighboring N-BAR proteins on vesicles but not on tubes. Furthermore, our model predicts that, for deep immersion of the H0 helices, the interplay between H0-induced leaflet curvature and thickness deformations yields an optimal H0 separation. In particular, for the parameter values used for Fig. 3 we find an optimal H0 separation *d* ≈ 2 nm for *U* ≈ −0.9 nm. While available experimental data does not allow a precise estimate of the separation of neighboring H0 helices in supramolecular N-BAR chains, such a characteristic H0 separation is consistent with EPR experiments on tube-bound N-BAR proteins^22,23^. Finally, Fig. 3 also predicts that a transition from deep to shallow wedging of the H0 helices in N-BAR chains yields a transition from favorable to unfavorable H0-induced N-BAR interactions, which may assist in the disassembly of N-BAR chains^27^. Such a change in H0 membrane immersion depth could potentially be accomplished through phosphorylation of N-BAR proteins^22^. In agreement with electron cryo-tomography experiments^5,27,33^, the predicted bilayer-mediated N-BAR interactions due to the H0 helices are highly pliable, nonspecific, and can stabilize loose assemblies of N-BAR proteins without requiring direct protein-protein contacts between N-BAR proteins, which could potentially allow the formation of mixed N-BAR chains consisting of, for instance, amphiphysin and endophilin^5^.

### Bilayer-thickness-mediated H0 interactions facilitate self-assembly of N-BAR chains

To test whether, if one allows for arbitrary configurations of neighboring N-BAR proteins and boundary effects arising from the H0 tips, lipid bilayer-mediated interactions between N-BAR proteins due to H0-induced leaflet thickness deformations can yield self-assembly of extended tip-to-tail N-BAR chains^5,12,26–28,33^, we performed Monte Carlo simulations with simulated annealing of N-BAR pair potentials. We note that simulated annealing Monte Carlo simulations are an approach for finding low-energy configurations of a system, and hence do not capture thermal effects or effects depending on the concentration of N-BAR proteins. We modeled N-BAR proteins as rigid molecules formed by two H0 helices joined together by a protein backbone representing the BAR domain (see Fig. 4A), with arbitrary separations and orientations of N-BAR proteins. We consider, for now, interactions between N-BAR proteins arising solely from leaflet thickness deformations induced by the H0 helices with *U* ≈ −0.9 nm^22,23^, which we computed from equation (3) with *h*^+^ = 0 using finite elements^40^ and which are approximately pairwise additive (see Supplementary Information Sec. S6). In agreement with experiments^5,12,26–28,33^, and as expected from Fig. 3, we find that, starting from a random initial distribution of N-BAR proteins, N-BAR interactions due to H0-induced leaflet thickness deformations yield self-assembly of extended tip-to-tail N-BAR chains via formation of antiparallel dimers of the H0 helices of neighboring N-BAR proteins (Fig. 4A). The nonspecificity of H0-induced N-BAR interactions implied by our model, and also suggested by experiments^5,27,33^, thereby results in a degenerate architecture of antiparallel H0 dimers in N-BAR chains, with favorable links in N-BAR chains along either one of the two elongated sides of the H0 helices of neighboring N-BAR proteins (Fig. 4A).

EPR experiments^22,23^ have shown that, for N-BAR proteins with the H0 helices in the deep immersion state *U* ≈ −0.9 nm, the BAR scaffolding domain is in contact with the lipid bilayer. The resulting BAR-induced membrane curvature deformations have been studied extensively within the Helfrich-Canham-Evans framework of membrane elasticity^19–21,51^. In particular, the contributions to bilayer-mediated N-BAR interactions due to the BAR domain have been found to favor the face-on orientation of neighboring N-BAR proteins^52–54^. In contrast, we find here that the N-BAR interactions due to the H0 helices yield the observed tip-to-tail arrangement of neighboring N-BAR proteins^5,12,26–28,33^. To test whether contributions to N-BAR interactions due to the BAR domain may disrupt the tip-to-tail N-BAR chains favored by bilayer-thickness-mediated H0 interactions, we allowed for contributions to N-BAR interactions due to the BAR domain as well as H0-induced leaflet thickness deformations (see Fig. 4B). We thereby employed a BAR pair interaction potential based on previous calculations^53^ of bilayer-mediated interactions between endophilin BAR domains (see the Methods section). While the contributions to bilayer-mediated N-BAR interactions due to the BAR domain are not expected to be pairwise additive^69^, it has been found^52–54^ that BAR pair potentials can still provide useful insights into self-assembly of protein clusters via bilayer-mediated BAR interactions. The total pair interaction potential employed for Fig. 4B is given by the sum of the BAR interactions^53^ and the N-BAR interactions due to H0-induced bilayer thickness deformations also used for Fig. 4A. We note that membrane curvature only indirectly enters Fig. 4B through the effective BAR pair interactions^53^, and that the generic properties of bilayer-mediated H0 interactions are not sensitive to membrane curvature (see Supplementary Information Sec. S5). Figure 4B shows that, if one allows for N-BAR interactions due to the BAR domains as well as the H0 helices, bilayer-thickness-mediated H0 interactions still yield self-assembly of tip-to-tail N-BAR chains, but that the degeneracy in the architecture of antiparallel H0 dimers is lifted. Consistent with experimental observations^5,26,27^, our simulations suggest that N-BAR configurations with the “outer” elongated sides of the H0 helices of neighboring N-BAR proteins facing each other provide the most favorable links in supramolecular N-BAR chains. Furthermore, our results suggest that the interplay of H0-induced leaflet thickness deformations and bilayer-BAR interactions^52–54^ can yield self-assembly of supramolecular N-BAR chains into extended two-dimensional lattices.

### Membrane tension weakens bilayer-thickness-mediated H0 interactions

*In vitro* experiments^30^ have shown that a finite membrane tension decreases the propensity of endophilin to tubulate large vesicles. While the observed effect of membrane tension on curvature generation by endophilin may have a variety of different origins^30,32^, MD simulations^32^ have suggested that membrane tension inhibits self-assembly of supramolecular endophilin chains. Proceeding as for Fig. 3, we used equation (3) to explore how membrane tension modifies bilayer-mediated N-BAR interactions due to the H0 helices (see Fig. 5). In particular, we focus in Fig. 5 on H0 helices in the face-on orientation and neglect boundary effects due to the H0 tips. We find that, for the deep H0 immersion state *U* ≈ −0.9 nm^22,23^, an increased membrane tension makes self-assembly of supramolecular N-BAR chains via dimerization of neighboring H0 helices^5,12,26–28^ less favorable. This result can be understood by noting that a finite membrane tension tends to reduce the preferred thickness of the lipid bilayer, thus diminishing the effect of H0-induced leaflet thickness deformations. Consistent with this picture, we find that membrane tension only has a minor effect on H0-induced N-BAR interactions for the shallow H0 immersion state *U* ≈ 0 nm^22,23^, for which the leaflet curvature deformations induced by the H0 helices dominate over leaflet thickness deformations. However, equation (3) implies that, compared to changes in H0 immersion depth (Fig. 3), the H0 dimerization energy does not have a pronounced dependence on membrane tension, with shifts in dimerization energy <1 *k*_*B*_*T* for the typical membrane tensions encountered in experiments^30^.

### Concerted structural reorganization of N-BAR proteins

Equation (3) predicts that bilayer-mediated H0 interactions depend strongly on the separation of the H0 helices of neighboring N-BAR proteins as well as on the H0 membrane immersion depth (Fig. 3). This implies that, for large enough concentrations of membrane-bound N-BAR proteins, the energy difference between the observed N-BAR structures with deep and shallow immersion of the H0 helices^22,23^ involves a cooperative component due to H0-induced interactions between N-BAR proteins. EPR experiments^22,23^ indicate that, at low N-BAR concentrations, N-BAR proteins show shallow wedging of the H0 helices. Figure 3 predicts that H0-induced interactions between N-BAR proteins are unfavorable for the shallow immersion state of the H0 helices but can be favorable for the deep immersion state of the H0 helices^22,23^. We therefore speculate that, at high enough N-BAR concentrations, H0-induced N-BAR interactions may contribute to a cooperative transition of N-BAR proteins from a conformational state with shallow immersion of the H0 helices to a conformational state with deep immersion of the H0 helices^13,14,22–24^.

We illustrate the above physical mechanism for the concerted structural reorganization of N-BAR proteins^13,14,22–24^ via H0-induced N-BAR interactions by considering a simplified system consisting of a pair of diffusing, membrane-bound N-BAR proteins that interact via the H0-induced lipid bilayer deformations captured by equation (3). We thereby assume that the N-BAR concentration is low enough so that three or more N-BAR proteins are unlikely to be in close spatial proximity. Furthermore, we focus on N-BAR configurations for which the H0 helices of the two neighboring N-BAR proteins face each other, which is expected to provide a particularly favorable N-BAR configuration for a cooperative transition to the deep immersion state of the H0 helices (Figs. 3 and 4), and neglect any boundary effects due to the tips of the H0 helices. We specify the collective state of the N-BAR proteins through the variables *d*, *s*_1_, and *s*_2_, where *s*_1,2_ = 0,1 for the observed N-BAR conformations with shallow and deep immersion of the H0 helices^22,23^. The total energy of the pair of N-BAR proteins, *G*_pair_, can be expressed as

4 $${G}_{{\rm{pair}}}({s}_{1},{s}_{2},d)={G}_{{\rm{non}}}({s}_{1},{s}_{2})+{G}_{{\rm{int}}}({s}_{1},{s}_{2},d),$$

where *G*_non_ is the energy of the system in the absence of any interactions between the two N-BAR proteins, *d* → ∞, and *G*_int_ is the N-BAR pair interaction potential due to the H0 helices. Without loss of generality, we set *G*_non_ (0,0) = 0 and write

5 $${G}_{{\rm{non}}}({s}_{1},{s}_{2})=({s}_{1}+{s}_{2}){\varepsilon }_{d},$$

where *ε*_*d*_ is the energy difference between the N-BAR conformations with deep and shallow immersion of the H0 helices^22,23^ at *d* → ∞. The value of the parameter *ε*_*d*_ is determined by internal conformational changes of the N-BAR proteins as well as the interactions between the lipid bilayer and the N-BAR proteins at *d* → ∞. As mentioned above, EPR experiments indicate that low N-BAR concentrations favor *s*_1,2_ = 0^22,23^, suggesting that *ε*_*d*_ > 0. In the absence of detailed numerical estimates of *ε*_*d*_, we regard *ε*_*d*_ as a free parameter. We analytically calculate *G*_int_ from equation (3) following similar steps as for Fig. 3 (see the Methods section).

From equation (4), the Boltzmann weight associated with a state (*s*_1_, *s*_2_), sampled over *d*, can be estimated as (see Supplementary Information Sec. S7)^70^

6 $$z({s}_{1},{s}_{2})={e}^{-{G}_{{\rm{non}}}({s}_{1},{s}_{2})}\,[1+c\pi {\int }_{2{r}_{0}}^{{r}_{c}}{\rm{d}}r\,{f}_{12}({s}_{1},{s}_{2},r)r],$$

where *c* = 2/*A* denotes the concentration of membrane-bound N-BAR proteins, with *A* corresponding to the average membrane area occupied by two N-BAR proteins, *r*_*c*_ is the long-range cutoff for pair interactions, and the Mayer-*f* function

7 $${f}_{12}({s}_{1},{s}_{2},r)\equiv {e}^{-{G}_{{\rm{int}}}({s}_{1},{s}_{2},r)}-1$$

captures contributions to the conformational statistics of N-BAR proteins due to H0-induced N-BAR interactions. Equation (6) allows calculation of the probability that the pair of N-BAR proteins is collectively in the observed conformational state with deep immersion of the H0 helices^22,23^:

8 $${P}_{d}=\frac{z\mathrm{(1,1)}}{z\mathrm{(0,0)}+2z\mathrm{(0,1)}+z\mathrm{(1,1)}}.$$

To evaluate equation (8) we allowed for pair interactions up to a H0 axis-to-axis separation *r*_*c*_ = 40 nm in equation (6), which is well beyond the range over which the H0 helices of N-BAR proteins show strong interactions (Fig. 3). For *ε*_*d*_ > 0, contributions to equation (8) due to *G*_non_ bias the system towards N-BAR states with shallow immersion of the H0 helices. In contrast, contributions to equation (8) due to *G*_int_ tend to favor N-BAR states with deep immersion of the H0 helices^22,23^, with the competition between *G*_non_ and *G*_int_ governed by *c* (see Fig. 6). Equation (8) thus predicts that, as the N-BAR concentration is increased to a threshold concentration, bilayer-mediated N-BAR interactions due to the H0 helices can yield a concerted structural reorganization of N-BAR proteins from the observed conformational state with shallow wedging of the H0 helices to the observed conformational state with deep wedging of the H0 helices^22,23^, with the latter state favoring self-assembly of supramolecular N-BAR chains^5,12,26–28,33^. Additional cooperative contributions to the transition energy between the N-BAR states with shallow and deep membrane immersion of the H0 helices^22,23^ may originate from, for instance, bilayer-mediated N-BAR interactions due to the BAR domains^52–54,69^.

## Discussion

EPR experiments have demonstrated a conformational switch in N-BAR proteins^22,23^, with shallow membrane immersion of the H0 helices on vesicles but deep immersion on tubes. In these experiments, the H0 membrane immersion depth was measured with respect to the unperturbed lipid bilayer thickness. Generation of vesicles by N-BAR proteins is thought to rely on membrane-bound N-BAR proteins being oriented in a range of different directions^22–24^. On tubes, in contrast, N-BAR proteins have been observed to assemble into supramolecular, locally ordered tip-to-tail chains with antiparallel alignment and dimerization of the H0 helices of neighboring N-BAR proteins^5,12,26–28,33^, which is thought to rely on nonspecific interactions between the H0 helices of N-BAR proteins. BAR proteins lacking the H0 helices show, on tubes, supramolecular configurations distinct from those of N-BAR proteins^2,5^. Motivated by these experimental observations, we have developed here a coarse-grained elastic model of bilayer-mediated wedge interactions that accounts for the local thinning of the lipid bilayer induced by amphipathic protein wedges. Our model is designed to predict, based on a minimal set of assumptions about detailed system properties, how bilayer-mediated wedge interactions depend on the wedge immersion depth. We find that the observed conformational switch in N-BAR proteins from shallow to deep wedging of the H0 helices^22,23^ yields a corresponding switch from unfavorable to favorable bilayer-mediated N-BAR interactions due to the H0 helices. For the shallow H0 wedging observed on vesicles^22,23^, our model predicts repulsive interactions between the H0 helices of neighboring N-BAR proteins, thus allowing a variety of different orientations of neighboring N-BAR proteins on vesicles. In contrast, for the deep H0 wedging observed on tubes^22,23^, our model predicts, in agreement with experiments^5,12,26–28,33^, self-assembly of locally ordered tip-to-tail N-BAR chains via antiparallel alignment and dimerization of the H0 helices of neighboring N-BAR proteins.

We find here that, as the N-BAR concentration is increased to a threshold concentration, bilayer-mediated interactions between the H0 helices of N-BAR proteins can yield a concerted structural reorganization of N-BAR proteins from the observed conformational state with shallow wedging of the H0 helices to the observed conformational state with deep wedging of the H0 helices^22,23^. We therefore speculate that, as the N-BAR concentration is increased, H0-induced N-BAR interactions may contribute to a collective transition of N-BAR proteins from a conformational state with shallow membrane immersion of the H0 helices to a conformational state with deep membrane immersion of the H0 helices^13,14,22–24^. Combined with previous models of bilayer-wedge^19,51,55–57^ and bilayer-scaffold^19–21,51–54^ interactions, our model of bilayer-mediated interactions between amphipathic protein wedges provides a unifying physical picture that identifies and links key features of the observed membrane-bound molecular structures^10,11,15–18,22,23^ and supramolecular configurations^5,12,22–24,26–28,31–33^ of N-BAR proteins. We have focused here on the H0 helices of N-BAR proteins. However, our model of bilayer-mediated wedge interactions is simple enough to only rely on generic features of bilayer-wedge interactions. Our results therefore suggest that, in analogy to integral membrane proteins^34–41^, wedge-induced lipid bilayer thickness deformations may provide a general physical mechanism contributing to the supramolecular organization of peripheral membrane proteins with amphipathic protein wedges. For instance, consistent with the results discussed here in the context of the H0 helices of N-BAR proteins, *α*-synuclein helices have been observed to cluster in areas of membrane remodeling^71^, and it has been found^72^ that a threshold concentration of islet amyloid polypeptide (IAPP or amylin) helices is required to initiate membrane remodeling. Since wedging of amphipathic helices is a common motif for bilayer-protein interactions^1–5^, bilayer-mediated wedge interactions may contribute to the supramolecular organization of membrane proteins in a variety of different settings, and provide a fundamental design principle for membrane organization independent of any specific protein-protein interactions.

## Methods

### Analytic solution of bilayer-mediated wedge interactions

We analytically solved the Euler-Lagrange equations associated with equation (3) to determine the stationary lipid bilayer deformation profile associated with equation (3) (see Supplementary Information Sec. S2). For one-dimensional systems with spatial coordinate *r* (Fig. 2), the resulting lipid bilayer deformation profile is given by

9 $${\bar{u}}^{+}(r)={A}_{0}{e}^{\sqrt{{\nu }^{+}}r}+{A}_{1}{e}^{-\sqrt{{\nu }^{+}}r}+{A}_{2}{e}^{\sqrt{{\nu }^{-}}r}+{A}_{3}{e}^{-\sqrt{{\nu }^{-}}r},$$

10 $${h}^{+}(r)=\frac{{\bar{u}}^{+}(r)}{2}+{B}_{0}+{B}_{1}r+{B}_{2}{e}^{r/{\lambda }_{t}}+{B}_{3}{e}^{-r/{\lambda }_{t}},$$

where ${\bar{u}}^{+}={u}^{+}+\tau a/{K}_{t}$ and the inverse square decay length

11 $${{\rm{\nu }}}^{\pm }=\frac{1}{2}[{\lambda }_{t}^{-2}\pm {({\lambda }_{t}^{-4}-{\lambda }_{s}^{-4})}^{\mathrm{1/2}}],$$

with the characteristic length scales ${\lambda }_{t}={({K}_{b}/\tau )}^{\mathrm{1/2}}$ and ${\lambda }_{s}={({K}_{b}{a}^{2}/8{K}_{t})}^{1/4}$ arising from the competition, in equation (3), between the leaflet bending terms and the tension and leaflet thickness deformation (Hookian spring) terms, respectively.

To determine from equations (9) and (10) the energy potential for bilayer-mediated wedge interactions we proceeded in analogy to the classic elastic model of bilayer-protein interactions for integral membrane proteins^34–36,40,64–66^, and fixed the coefficients *A*_*i*_ and *B*_*j*_ with *i* = 0, 1, 2, 3 and *j* = 0, 1, 2, 3 in equations (9) and (10) from the boundary conditions on *h*^+^(*r*) and *u*^+^(*r*), and their derivatives, for the lipid bilayer region separating the two protein wedges (see Supplementary Information Secs. S2 and S3): Since equation (3) is invariant under constant shifts in *h*^+^(*r*), we set the reference value of *h*^+^(*r*) equal to zero without loss of generality, and minimized equation (3) with respect to the height difference of the two wedges, which amounts to imposing zero vertical force on the protein wedges (see Supplementary Information Appendix A). Furthermore, we set *u*^+^(*r*) = *U* along the bilayer-wedge interfaces, with *U* ≈ 0 and *U* ≈ −0.9 nm for the shallow and deep H0 immersion states of N-BAR proteins^22,23^. Based on the helical wheel of the H0 helices of endophilin^22^, a computational model of bilayer-wedge interactions^19,51,55,57^, and previous studies of bilayer-protein interactions for integral membrane proteins^36,40,64,73^ we imposed slopes of *h*^+^ and *u*^+^ equal to −tan 9° and 0 along the bilayer-wedge interfaces, but our key model predictions are robust with respect to variations in these parameter values (see Supplementary Information Sec. S3). We note that, in the context of integral membrane proteins, the boundary conditions on the slope of the lipid bilayer thickness deformations at the bilayer-protein interface have been examined in some detail using MD simulations^73,74^, with excellent agreement between MD simulations and continuum elastic models for small (but finite) contact slopes of the bilayer thickness deformations^73^. We analytically calculated the wedge interaction potential used for Figs 3, 5 and 6 by transforming equation (3) with equations (9) and (10) into the form

12 $$\begin{array}{c}G=L\int {\rm{d}}r\{\nabla [\frac{{K}_{b}}{2}(\nabla {h}^{+}{\nabla }^{2}{h}^{+}-{h}^{+}{\nabla }^{3}{h}^{+}-\frac{1}{2}\nabla {h}^{+}{\nabla }^{2}{\bar{u}}^{+}+\frac{1}{2}{h}^{+}{\nabla }^{3}{\bar{u}}^{+}+\frac{1}{2}\nabla {\bar{u}}^{+}{\nabla }^{2}{\bar{u}}^{+}-\frac{1}{2}{\bar{u}}^{+}{\nabla }^{3}{\bar{u}}^{+}\\ \,\,+\frac{1}{2}{\bar{u}}^{+}{\nabla }^{3}{h}^{+}-\frac{1}{2}\nabla {\bar{u}}^{+}{\nabla }^{2}{h}^{+})+\frac{\tau }{2}({h}^{+}\nabla {h}^{+}-\frac{1}{2}{h}^{+}\nabla {\bar{u}}^{+}+\frac{1}{2}{\bar{u}}^{+}\nabla {\bar{u}}^{+}-\frac{1}{2}{\bar{u}}^{+}\nabla {h}^{+})]-\frac{{\tau }^{2}}{4{K}_{t}}\},\end{array}$$

where $\nabla \equiv \frac{{\rm{d}}}{{\rm{d}}r}$, applying Gauss’s theorem, and subtracting from equation (12) the contribution to *G* not due to interactions, ${G}_{0}={\mathrm{lim}}_{d\to \infty }G(d)$. Detailed numerical estimates of the magnitude of bilayer-mediated wedge interactions, and the characteristic wedge separations favored by bilayer-mediated wedge interactions, may require a closer examination of the boundary conditions imposed on the slopes of *u*^+^ and *h*^+^ at the bilayer-wedge interfaces, and quantification of the relation between the measured wedge immersion depths^22,23^ and the wedge-induced lipid bilayer thickness deformations in the immediate vicinity of protein wedges.

### Simulated annealing Monte Carlo simulations

For Fig. 4, we computed the directional N-BAR pair potentials due to H0-induced leaflet thickness deformations in two-dimensional membranes by minimizing equation (3) for *h*^+^ = 0 using finite elements as described previously^40^. Along the extended sides of the H0 helices we imposed a constant *U* ≈ −0.9 nm corresponding to the deep immersion state of the H0 helices of N-BAR proteins^22,23^, and gradually let *U* → 0 along the (rounded) wedge tips towards the centers of the wedge tips. For Fig. 4B we also used, based on previous calculations of bilayer-mediated interactions between endophilin BAR domains^53^, the modified Lennard-Jones pair potential

13 $${G}_{{\rm{int}}}^{{\rm{BAR}}}(r,{\theta }_{1},{\theta }_{2})=\varepsilon [{(\frac{{r}_{m}}{r})}^{12}-2{(\frac{{r}_{m}}{r})}^{6}(1-\frac{\mathrm{|2}{\theta }_{1}-\pi |}{2\pi }-\frac{\mathrm{|2}{\theta }_{2}-\pi |}{2\pi })]\,,$$

where *r* is the center-to-center distance between BAR domains, the angles *θ*_1, 2_ capture the BAR orientations with a positive modulus *π* rad (with the BAR domains facing each other for *θ*_1_ = *θ*_2_ = *π*/2), *ε*_*d*_ = 10 *k*_*B*_*T*, and *r*_*m*_ = 10 nm. For the simulated annealing Monte Carlo simulations in Fig. 4, a single Monte Carlo step consisted, on average, of one displacement trial (*δr* = 0.1 nm) and one rotation trial (*δθ* = 1°) per N-BAR protein. The trials were accepted or rejected according to the Metropolis algorithm. In a typical run, we first used 10^7^ Monte Carlo steps at constant *T* = 2*T*_rm_, where *T*_rm_ = 298 K is the room temperature, and then decreased the temperature linearly to *T* = 0 over 5 × 10^6^ Monte Carlo steps. (See Supplementary Information Sec. S6 for further details).

## Electronic supplementary material

Supplementary Information

## Data Availability

All data generated or analyzed as part of this study are available through this published Article (and its Supplementary Information).
