# Supplementary material for: Directed Supramolecular Organization of N-BAR Proteins through Regulation of H0 Membrane Immersion Depth
Source: Sci Rep. 2018 Nov 6;8:16383. doi: 10.1038/s41598-018-34273-2 (PMC6219572; doi:10.1038/s41598-018-34273-2)
Supplement: Supplementary file 1 — Supplementary Information [file 41598_2018_34273_MOESM1_ESM.pdf]

Directed Supramolecular Organization of N-BAR Proteins through Regulation of H0 Membrane Immersion Depth

Supplementary Information

Osman Kahraman[1, 2], Ralf Langen[3], and Christoph A. Haselwandter[1]

[1] *Department of Physics & Astronomy and Molecular and Computational Biology Program, Department of Biological Sciences, University of Southern California, Los Angeles, CA 90089, USA*

[2] *Present address: R&D Center, Arcelik A.S., Tuzla, Istanbul, 34950, Turkey*

[3] *Department of Biochemistry and Molecular Biology, Zilkha Neurogenetic Institute, Keck School of Medicine, University of Southern California, Los Angeles, CA 90033, USA*

S1 Elastic model of wedge-induced bilayer thickness deformations

Peripheral membrane proteins inserting partially into one lipid bilayer leaflet are expected [1–5] to induce shape and/or thickness deformations in one lipid bilayer leaflet while only indirectly affecting the other bilayer leaflet via the coupling between the two leaflets. A physical model describing the lipid bilayer deformations induced by protein wedges must therefore be able to account for protein-induced deviations from the preferred membrane shape and thickness in one lipid bilayer leaflet without requiring direct lipid-protein interactions in the other bilayer leaflet. The classic continuum elasticity theory of protein-induced lipid bilayer deformations [6–10] successfully accounts for the symmetric lipid bilayer thickness deformations induced by integral proteins spanning the lipid bilayer. Recent generalizations of the continuum elasticity theory of lipid bilayer membranes [11–17] allow for asymmetric lipid bilayer deformations and are thus suitable for capturing bilayer-wedge interactions.

To account for the basic phenomenology of bilayer-wedge interactions, we follow here the formalism developed by Bitbol *et al.* [14] and describe wedge-induced lipid bilayer deformations using four fields, $h^{\pm}(x,y)$ and $u^{\pm}(x,y)$, capturing height and thickness deformations in the upper (+) and lower (−) lipid bilayer leaflets, respectively (see Fig. 2 of the main text). Expanding the lipid bilayer elastic energy, G_{bilayer}, in terms of the lipid area deformations and the leaflet curvatures up to second order one finds [14]

$$G_{\mathrm{bilayer}} = \int \mathrm{d}x\mathrm{d}y \left(f_0^+ + f_0^-\right) , \tag{S1}$$

where the upper and lower leaflet energy densities, $f_0^{\pm}$, are given by

$$\begin{aligned} f_0^{\pm} =& \frac{\tau}{2}\left(1 + \frac{u^{\pm}}{a} + \frac{(\nabla h^{\pm})^2}{2}\right) + \frac{K_t}{4a^2}(u^{\pm})^2 + \frac{K_b}{4}(\nabla^2 h^{\pm})^2 \pm \frac{K_b C_0}{2}\nabla^2 h^{\pm} \pm \frac{K_b}{2a}(C_0 - C_0')u^{\pm}\nabla^2 h^{\pm} \\ &+ \frac{K_G}{2}\det(\partial_i\partial_j h^{\pm}) + k_a'(\nabla u^{\pm})^2 + 4k_a'' a^2 (\nabla^2 u^{\pm})^2 + \beta\left[\nabla\cdot(u^{\pm}\nabla u^{\pm}) - a\nabla^2 u^{\pm}\right] , \end{aligned} \tag{S2}$$

where τ is the membrane tension, a is the thickness of the unperturbed monolayer hydrophobic core, K_t is the thickness deformation modulus, K_b is the bending rigidity, K_G is the Gaussian bending rigidity, C_0 is the intrinsic (spontaneous) curvature, C_0' captures the modification of the intrinsic curvature due to area variations, and the terms k_a', k_a'', and β arise from the first- and second-order variations in the area per lipid and have the same dimension as the membrane tension. Since the two lipid bilayer leaflets form a continuous bilayer with no overlaps or voids, the four fields entering Eq. (S1) must satisfy a continuity constraint. To second order, this continuity constraint is given by [14]

$$h^+ - h^- = \left(u^+ + a\right)\left[1 - \frac{(\nabla h^+)^2}{2}\right] + \left(u^- + a\right)\left[1 - \frac{(\nabla h^-)^2}{2}\right] , \tag{S3}$$

which, to leading order, reduces to Eq. (2) of the main text.

For our minimal elastic model of bilayer-mediated wedge interactions we simplify the general model in Eq. (S1), as follows. First, we focus on bilayers with vanishing intrinsic curvature, yielding $C_0 = 0$ and $C_0' = 0$ in Eq. (S1). Second, we note that the terms in Eq. (S1) with K_G and β as coefficients enter the energy of wedge-induced lipid bilayer deformations only as boundary terms. Such boundary terms are expected to contribute to the insertion energy of N-BAR proteins, as well as the energy difference between different membrane-bound conformational states of N-BAR proteins [1,2] (hence contributing to the effective parameter ϵ_d in the main text). However, for a given N-BAR state (and membrane topology), these terms do not affect bilayer-mediated interactions between N-BAR proteins due to the H0 helices. We therefore set $K_G = 0$ and $\beta = 0$ in Eq. (S1). Third, the coefficients k_a' and k_a'' in Eq. (S1) modify how the membrane tension and bilayer bending rigidity couple to leaflet thickness deformations. It has been argued [14] that $k_a'' \approx 0$ but that k_a' may produce a considerable increase in the effective value of the membrane tension, which could make the dependence of H0-induced N-BAR interactions on membrane tension more pronounced. However, it has also been found, in the case of integral membrane proteins, that the term with coefficient k_a' in Eq. (S1) is not necessary for a continuum elastic theory of the form in Eq. (S1) to successfully predict the basic experimental phenomenology of bilayer-protein interactions [7, 9, 18–24] and to yield excellent agreement with the results of MD simulations [25]. We therefore set here $k_a' = 0$ and $k_a'' = 0$, while noting that detailed numerical estimates of the magnitude of the bilayer-mediated N-BAR interactions induced by the H0 helices, and the resulting optimal H0 separations, may require inclusion of these terms. Finally, we neglect the terms in Eq. (S1) that are independent of the membrane deformation fields, since such terms do not contribute to the energy cost of wedge-induced lipid bilayer deformations. Equation (S1)

thus reduces to

$$G = \frac{1}{2}\int \mathrm{d}x\mathrm{d}y\left\{\frac{K_b}{2}\left[\left(\nabla^2 h^+\right)^2 + \left(\nabla^2 h^-\right)^2\right] + \frac{K_t}{2}\left[\left(\frac{u^+}{a}\right)^2 + \left(\frac{u^-}{a}\right)^2\right]\right.$$
$$\left. + \tau\left[\frac{u^+}{a} + \frac{u^-}{a}\right] + \frac{\tau}{2}\left[(\nabla h^+)^2 + (\nabla h^-)^2\right]\right\}, \quad \text{(S4)}$$

which is equivalent to Eq. (1) of the main text.

As noted in the main text, insertion of amphipathic protein wedges can substantially decrease the thickness of lipid bilayers [1–4, 26–32]. In the case of symmetric bilayer thickness deformations, $u^+ = u^-$, Eq. (S4) with Eq. (S3) reduces to the classic elastic model of bilayer-protein interactions for integral membrane proteins [5,7,9,18–24,33]. In this model, bilayer thickness and bilayer midplane deformations mathematically decouple from each other to leading order. The model predicts (strongly) favorable bilayer-mediated protein interactions at small protein separations, yielding a general physical mechanism for the organization of integral membrane proteins [5, 9, 18–22, 33]. In contrast, amphipathic protein wedges only directly interact with one lipid bilayer leaflet, which we take to correspond to the upper leaflet (see Fig. 2 of the main text). As a result, protein wedges are expected [1–5] to asymmetrically perturb the lipid bilayer with, for large enough wedge immersion depths, substantial compression of the leaflet into which the wedge is inserted, but only minor leaflet thickness deformations in the opposing leaflet. In analogy to the assumption of hydrophobic matching underlying bilayer-mediated interactions between integral membrane proteins [5,9,18–22,33], it is thereby assumed that the upper lipid bilayer leaflet compresses so as to maintain favorable lipid-wedge interactions as the wedge immersion depth is increased [1, 2]. While Eq. (S4) can account for deformations in both u^+ and u^-, we thus simplify our model by assuming weak leaflet thickness deformations in the lower leaflet, and set $u^- = 0$. Equation (S4) then reduces to Eq. (3) of the main text,

$$G = \frac{1}{2}\int \mathrm{d}x\mathrm{d}y\left\{K_b\left[\left(\nabla^2 h^+\right)^2 - \left(\nabla^2 h^+\right)\left(\nabla^2 u^+\right) + \frac{1}{2}\left(\nabla^2 u^+\right)^2\right]\right.$$
$$\left. + \frac{K_t}{2}\left(\frac{u^+}{a}\right)^2 + \tau\frac{u^+}{a} + \tau\left[\left(\nabla h^+\right)^2 - \left(\nabla h^+\right)\cdot\left(\nabla u^+\right) + \frac{1}{2}\left(\nabla u^+\right)^2\right]\right\}, \quad \text{(S5)}$$

which we employed for all the calculations described here. Note that, via Eq. (S3), the field h^- is completely specified by u^+ and h^+ which, in turn, are determined through minimization of Eq. (S5) with respect to u^+ and h^+. From an intuitive perspective, and in analogy to integral membrane proteins [5, 7, 9, 18–24, 33], the field u^+ and its derivatives represent wedge-induced deformations of the bilayer hydrophobic thickness from its unperturbed (flat) state, while the (2nd-order) derivatives of h^+ can be viewed as representing wedge-induced curvature deformations of the lipid bilayer from its unperturbed (flat) state. Contrary to integral membrane proteins, however, the elastic energies governing u^+ and h^+ do not decouple from each other in Eq. (S5).

The model in Eq. (S5) extends [12–14, 17] the classic coarse-grained models of bilayer-protein interactions for integral membrane proteins [5, 7, 9, 18–24, 33] and protein scaffolds [34–40] to amphipathic protein wedges. Following previous studies [1–4, 26–37, 41–45], Eq. (S5) assumes that amphipathic protein wedges such as the H0 helices of N-BAR proteins can deform the lipid bilayer shape and thickness. The simplicity of our model means that our results only rely on generic features of bilayer-wedge interactions, and may therefore apply to a variety of different systems [41–45]. However, the simplicity of our model also means that it does not capture the detailed molecular properties of bilayer-wedge interactions. For instance, wedges may induce lipid tilt. At the cost of introducing additional assumptions about how wedge-induced lipid tilt deformations depend on the key structural parameters characterizing bilayer-wedge interactions [1, 2, 34, 35, 46–50], wedge-induced lipid tilt deformations could be included in the framework employed here [15–17, 51, 52]. In the case of integral membrane proteins, previous work has shown that protein-induced lipid bilayer shape and thickness deformations are sufficient to explain the observed clustering of a variety of proteins [5, 9, 18–22, 33], to successfully predict the basic experimental phenomenology of bilayer-protein interactions [7, 9, 18–24], and to produce excellent agreement with the results of MD simulations on protein-induced lipid bilayer deformations [25], without invoking additional molecular degrees of freedom such as lipid tilt deformations. It remains to be seen under what circumstances lipid tilt deformations may play a crucial role in bilayer-mediated interactions between amphipathic protein wedges. Our model of bilayer-mediated wedge interactions is not designed to allow prediction of the effective intrinsic curvature produced by protein wedges at the bilayer-wedge interface, which can be obtained from complementary modeling approaches [34, 35, 46–49] (see Sec. S3).

S2 Analytic solution of bilayer-mediated wedge interactions

In this section we focus on the most straightforward scenario of two H0 helices facing each other. In this case, the system can be approximated as being effectively one-dimensional, with the spatial coordinate r being measured perpendicular to the (projected) helix axes (see Fig. S1). We denote the left and right helices by "wedge 1" and "wedge 2," respectively. For convenience we choose the origin $r = 0$ of the coordinate system to be equidistant from the two helix axes, i.e., the two helix axes are located at $r = \pm d/2$. We denote the helix radius by r_0 and the helix length by L. In our one-dimensional model, the bilayer-mediated wedge interactions are set by the wedge-induced deformations of the lipid bilayer region separating the two wedges, which corresponds to $-d/2 + r_0 < r < d/2 - r_0$. In the following we provide the exact analytic solution of the interaction potential between H0 helices for this effectively one-dimensional system.

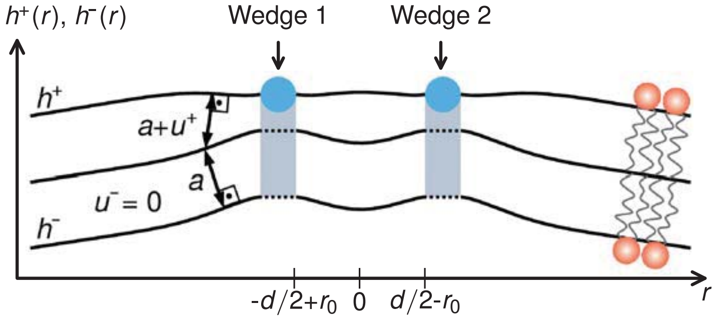

Figure S1: Schematic of the coordinate system used for the analytic calculation of the interaction potential between protein wedges in the face-on configuration (see also Fig. 2 of the main text). We approximate the system as being effectively one-dimensional, with the only relevant spatial dimension for bilayer-mediated wedge interaction being the distance d separating neighboring amphipathic helices perpendicular to the helix axes. The schematic shows a cross section of the system perpendicular to the helix axes. The function $h^-(r)$ is completely specified by the functions $h^+(r)$ and $u^+(r)$ according to Eq. (S3) with $u^- = 0$.

Replacing the fields $h^+(x,y)$ and $u^+(x,y)$ in Eq. (3) of the main text by the fields $h^+(r)$ and $u^+(r)$ we obtain the coupled Euler-Lagrange equations (see Appendix A)

$$\begin{cases} K_b\left(2\frac{\mathrm{d}^4h^+}{\mathrm{d}r^4}-\frac{\mathrm{d}^4u^+}{\mathrm{d}r^4}\right)-\tau\left(2\frac{\mathrm{d}^2h^+}{\mathrm{d}r^2}-\frac{\mathrm{d}^2u^+}{\mathrm{d}r^2}\right)=0\,, \\ K_b\left(\frac{\mathrm{d}^4u^+}{\mathrm{d}r^4}-\frac{\mathrm{d}^4h^+}{\mathrm{d}r^4}\right)+\frac{K_t}{a^2}u^+-\tau\left(\frac{\mathrm{d}^2u^+}{\mathrm{d}r^2}-\frac{\mathrm{d}^2h^+}{\mathrm{d}r^2}\right)+\frac{\tau}{a}=0 \end{cases} \tag{S6}$$

specifying the extrema of Eq. (3) of the main text. To analytically solve Eq. (S6) we note that the first equation in Eq. (S6) can be used to eliminate the terms in the second equation in Eq. (S6) involving h^+. The solution of the system of differential equations in Eq. (S6) can therefore be obtained by solving, separately, two 4th-order ordinary differential equations. We thus find the general solution

$$\bar{u}^+(r)=A_0e^{\sqrt{\nu^+}r}+A_1e^{-\sqrt{\nu^+}r}+A_2e^{\sqrt{\nu^-}r}+A_3e^{-\sqrt{\nu^-}r}\,, \tag{S7}$$

$$h^+(r)=\frac{\bar{u}^+(r)}{2}+B_0+B_1r+B_2e^{r/\lambda_t}+B_3e^{-r/\lambda_t}\,, \tag{S8}$$

where, for mathematical convenience, we have introduced the function [21]

$$\bar{u}^+=u^++\frac{\tau a}{K_t} \tag{S9}$$

and defined an inverse square decay length

$$\nu^\pm=\frac{1}{2}\left[\lambda_t^{-2}\pm\left(\lambda_t^{-4}-\lambda_s^{-4}\right)^{1/2}\right]\,, \tag{S10}$$

with the characteristic length scales

$$\lambda_t = \left(\frac{K_b}{\tau}\right)^{1/2} \quad \text{and} \quad \lambda_s = \left(\frac{K_b a^2}{8K_t}\right)^{1/4} \tag{S11}$$

arising from the competition, in Eq. (3) of the main text, between the leaflet bending terms and the tension and leaflet thickness deformation (Hookian spring) terms, respectively.

The coefficients A_i and B_j with $i = 0,1,2,3$ and $j = 0,1,2,3$ in Eqs. (S7) and (S8) must be determined from the boundary conditions on the lipid bilayer. Following previous work on bilayer-protein interactions for integral membrane proteins [9,23,24], we first consider the boundary conditions on $h^+(r)$ and its derivative:

$$h^+\big|_{r=-d/2+r_0} = H_1\,, \quad h^+\big|_{r=d/2-r_0} = H_2\,, \tag{S12}$$

$$\left.\frac{\mathrm{d}h^+}{\mathrm{d}r}\right|_{r=-d/2+r_0} = H_1'\,, \quad \left.\frac{\mathrm{d}h^+}{\mathrm{d}r}\right|_{r=d/2-r_0} = H_2'\,, \tag{S13}$$

where $H_{1,2}$ and $H_{1,2}'$ are constants. Since Eq. (3) of the main text is invariant under constant shifts in h^+, we can set $H_1 = 0$ without loss of generality. We determine the value of H_2 by minimizing the energy in Eq. (3) of the main text with respect to H_2, resulting in

$$\left[K_b\left(2\frac{\mathrm{d}^3h^+}{\mathrm{d}r^3} - \frac{\mathrm{d}^3u^+}{\mathrm{d}r^3}\right) - \tau\left(2\frac{\mathrm{d}h^+}{\mathrm{d}r} - \frac{\mathrm{d}u^+}{\mathrm{d}r}\right)\right]_{r=d/2-r_0} = 0\,. \tag{S14}$$

The above two boundary conditions fixing $H_{1,2}$ amount [23,24] to imposing a condition of zero vertical force on the two protein wedges (see Appendix A). As expected [9, 23, 24], we find that Eq. (S14) yields $H_1 = H_2$ for identical wedges. If the wedges are, for instance, at different immersion depths, Eq. (S14) generally yields $H_1 \neq H_2$. If, instead of Eq. (S14), we do not allow H_2 to vary but assume that the two wedges are always fixed at the same height so that $H_1 = H_2$, we obtain similar results for bilayer-mediated wedge interactions as described in the main text. The values of $H_{1,2}'$ in Eq. (S13) are determined by the properties of the particular amphipathic protein wedge and lipid bilayer under consideration, a point we return to in Sec. S3. As noted in the main text, N-BAR proteins interact with lipid bilayers not just through a single H0 helix, but multiple interaction sites connected by a BAR domain. We therefore assume in Eqs. (S12) and (S13) that interactions between a given H0 helix and the remainder of the N-BAR protein prevent the H0 helix from tilting in response to bilayer-mediated wedge interactions [9,23,24]. For general protein wedges, which are not necessarily attached to a larger membrane-bound molecule, this assumption may not be warranted. In this case, Eqs. (S12) and (S13) would need to be modified to allow for a finite tilt angle, which can be obtained by imposing conditions of zero torque [23, 24] on the protein wedges (see Appendix A), and Eqs. (S12) and (S13) would need to be generalized to two-dimensional surfaces [23, 24] since one-dimensional systems are not expected to be able to capture curvature-mediated interactions between tilting protein wedges.

In analogy to Eqs. (S12) and (S13), and previous work on bilayer-protein interactions for integral membrane proteins [5, 7, 9, 21, 33], we obtain the remaining boundary conditions for the solution in Eqs. (S7) and (S8) by specifying $u^+(r)$ and its derivative along the bilayer-wedge interfaces (see Appendix A):

$$u^+\big|_{r=-d/2+r_0} = U_1\,, \quad u^+\big|_{r=d/2-r_0} = U_2\,, \tag{S15}$$

$$\left.\frac{\mathrm{d}u^+}{\mathrm{d}r}\right|_{r=-d/2+r_0} = U_1'\,, \quad \left.\frac{\mathrm{d}u^+}{\mathrm{d}r}\right|_{r=d/2-r_0} = U_2'\,, \tag{S16}$$

where $U_{1,2}$ and $U_{1,2}'$ are constants. As discussed in the main text, the wedge immersion depths $U_{1,2}$ (denoted by U in the main text) are key parameters for bilayer-mediated wedge interactions, which we estimate for the H0 helices of N-BAR proteins based on data obtained from EPR experiments [1,2]. In the context of integral membrane proteins, there has been some debate [5, 7, 11–14, 21, 25, 33, 53–58] as to whether the derivatives of the bilayer thickness deformation field along the bilayer-protein interface should be regarded as fixed, or whether the bilayer-protein interaction energy should be minimized with respect to these derivatives. We return to the values of $U_{1,2}'$ in Sec. S3.

Equations (S12)–(S16) allow us to determine the coefficients A_i and B_j in Eqs. (S7) and (S8) for the lipid bilayer region separating the two wedges and, hence, completely specify the analytic solution in Eqs. (S7) and (S8) for this region of the lipid bilayer. For convenience, we thereby numerically solve, for a given set of values of $H_{1,2}'$, $U_{1,2}$, and $U_{1,2}'$ (see Sec. S3), for the coefficients A_i and B_j in Eqs. (S7) and (S8). In Fig. S2 we show examples of the resulting wedge-induced lipid bilayer deformation profiles. Note that $H_1 = H_2 = 0$ for the two identical wedges considered in Fig. S2A and Fig. S2B, while Eq. (S14) yields $H_1 \neq H_2$ for the two wedges at distinct immersion depths considered in Fig. S2C. Figure S2 shows, in addition to the lipid bilayer region separating the two wedges, also the lipid bilayer regions extending from the two wedges to $r \to \pm\infty$. For these two solution domains, we use boundary conditions for the bilayer-wedge interfaces analogous to those used for the bilayer region separating the two wedges. We take the lipid bilayer to be flat as $r \to \pm\infty$ with all lipid bilayer deformations decaying to zero. In particular, we enforce

$$u^+\big|_{r=\pm\infty} = -\frac{\tau a}{K_t}\,, \quad \left.\frac{\mathrm{d}u^+}{\mathrm{d}r}\right|_{r=\pm\infty} = 0\,, \quad \left.\frac{\mathrm{d}h^+}{\mathrm{d}r}\right|_{r=\pm\infty} = 0\,, \tag{S17}$$

implying $A_1 = A_3 = B_1 = B_3 = 0$ for the lipid bilayer region extending to $r \to -\infty$, and $A_0 = A_2 = B_1 = B_2 = 0$ for the lipid bilayer region extending to $r \to \infty$. For the schematics in Fig. 2A of the main text and Fig. S1 we calculated the deformation profiles as in Fig. S2A with $d = 5$ nm. For the schematic in Fig. 2B of the main text we proceeded as for Fig. S2A but with $d = 2$ nm, and for the schematics in the insets of Fig. 3 of the main text we proceeded as for Figs. S2A and S2B but with $d = 3$ nm.

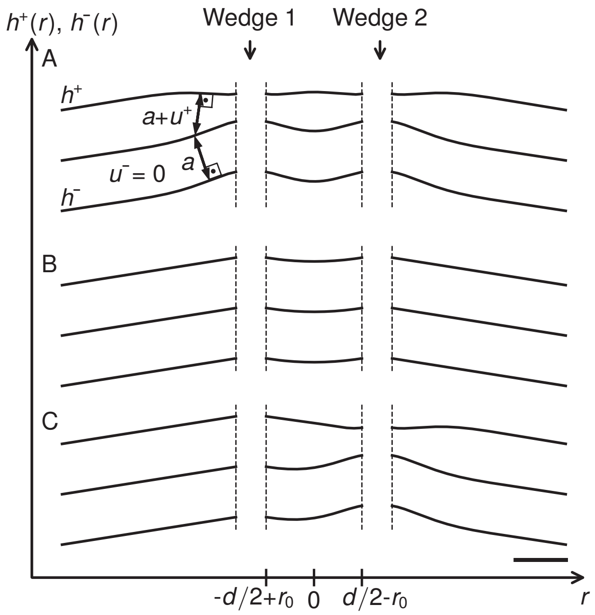

Figure S2: Wedge-induced lipid bilayer deformation profiles at $d = 5$ nm for (**A**) $U_1 = U_2 = -0.9$ nm, (**B**) $U_1 = U_2 = 0$ nm, and (**C**) $U_1 = 0$ nm and $U_2 = -0.9$ nm, obtained from the analytic solution in Eqs. (S7) and (S8), together with Eq. (2) of the main text, using the same parameter values as in the main text with $\tau = 0$. The boundaries of the lipid bilayer solution domains are indicated by dashed lines. Scale bar, 2 nm.

The general analytic solution in Eqs. (S7) and (S8) allows calculation of the energy of bilayer-mediated wedge interactions. It is thereby convenient to use the Euler-Lagrange equations in Eq. (S6) to transform the energy in Eq. (3) of the main text into the form

$$\begin{aligned} G =& L \int \mathrm{d}r \Bigg\{ \nabla \Bigg[\frac{K_b}{2} \Bigg(\nabla h^+ \nabla^2 h^+ - h^+ \nabla^3 h^+ - \frac{1}{2} \nabla h^+ \nabla^2 \bar{u}^+ + \frac{1}{2} h^+ \nabla^3 \bar{u}^+ + \frac{1}{2} \nabla \bar{u}^+ \nabla^2 \bar{u}^+ - \frac{1}{2} \bar{u}^+ \nabla^3 \bar{u}^+ \\ &+ \frac{1}{2} \bar{u}^+ \nabla^3 h^+ - \frac{1}{2} \nabla \bar{u}^+ \nabla^2 h^+ \Bigg) + \frac{\tau}{2} \Bigg(h^+ \nabla h^+ - \frac{1}{2} h^+ \nabla \bar{u}^+ + \frac{1}{2} \bar{u}^+ \nabla \bar{u}^+ - \frac{1}{2} \bar{u}^+ \nabla h^+ \Bigg) \Bigg] - \frac{\tau^2}{4K_t} \Bigg\}, \end{aligned} \tag{S18}$$

where, for simplicity, we focus on the effectively one-dimensional system considered here with $\nabla \equiv \frac{\mathrm{d}}{\mathrm{d}r}$. The integrand in Eq. (S18) can be transformed back into the integrand in Eq. (3) of the main text by noting that, in terms of $\bar{u}^+(r)$, the Euler-Lagrange equations in Eq. (S6) take the form

$$\begin{cases} E_1 \equiv K_b \left(2 \frac{\mathrm{d}^4 h^+}{\mathrm{d}r^4} - \frac{\mathrm{d}^4 \bar{u}^+}{\mathrm{d}r^4} \right) - \tau \left(2 \frac{\mathrm{d}^2 h^+}{\mathrm{d}r^2} - \frac{\mathrm{d}^2 \bar{u}^+}{\mathrm{d}r^2} \right) = 0\,, \\ E_2 \equiv K_b \left(\frac{\mathrm{d}^4 \bar{u}^+}{\mathrm{d}r^4} - \frac{\mathrm{d}^4 h^+}{\mathrm{d}r^4} \right) + \frac{K_t}{a^2} \bar{u}^+ - \tau \left(\frac{\mathrm{d}^2 \bar{u}^+}{\mathrm{d}r^2} - \frac{\mathrm{d}^2 h^+}{\mathrm{d}r^2} \right) = 0 \end{cases} . \tag{S19}$$

Equation (3) of the main text is then obtained by adding to Eq. (S18) the term

$$\frac{1}{4} \left[E_1 h^+(r) + E_2 \bar{u}^+(r) \right] , \tag{S20}$$

which is identical to zero for the analytic solution in Eqs. (S7) and (S8), and replacing $\bar{u}^+(r)$ by $u^+(r)$ via Eq. (S9).

We use Eq. (S18), together with Gauss's theorem, to analytically calculate the wedge interaction energy associated with the solution in Eqs. (S7) and (S8) by evaluating

$$\begin{aligned}G =& L\Bigg[\frac{K_b}{2}\bigg(\nabla h^+\nabla^2 h^+ - h^+\nabla^3 h^+ - \frac{1}{2}\nabla h^+\nabla^2\bar{u}^+ + \frac{1}{2}h^+\nabla^3\bar{u}^+ + \frac{1}{2}\nabla\bar{u}^+\nabla^2\bar{u}^+ - \frac{1}{2}\bar{u}^+\nabla^3\bar{u}^+ \\ &+ \frac{1}{2}\bar{u}^+\nabla^3 h^+ - \frac{1}{2}\nabla\bar{u}^+\nabla^2 h^+\bigg) + \frac{\tau}{2}\bigg(h^+\nabla h^+ - \frac{1}{2}h^+\nabla\bar{u}^+ + \frac{1}{2}\bar{u}^+\nabla\bar{u}^+ - \frac{1}{2}\bar{u}^+\nabla h^+\bigg)\Bigg]_{r=-d/2+r_0}^{r=d/2-r_0} .\end{aligned} \tag{S21}$$

Upon subtracting from the total energy of wedge-induced bilayer deformations in Eq. (S21) the contribution to G not due to interactions,

$$G_0 = \lim_{d\to\infty} G(d)\,, \tag{S22}$$

we obtain from Eq. (S21) the wedge interaction potential used for Figs. 3, 5, and 6 of the main text.

S3 Numerical values of model parameters

In our minimal elastic model of bilayer-mediated wedge interactions, the shape of the H0 helices of N-BAR proteins is specified by the H0 length L and the H0 radius r_0. As stated in the main text, we used the value $L \approx 3$ nm [50]. With an approximate helix length of 0.15 nm per amino acid (3.6 amino acids per turn of the α-helix, with a turn length ≈ 0.54 nm [59]), $L \approx 3$ nm yields a wedge length of approximately 20 amino acids. We used a H0 helix radius $r_0 \approx 0.6$ nm typical for α-helices [59]. EPR experiments [1, 2] suggest $U_{1,2} \approx 0$ nm for the shallow immersion state of the H0 helices of N-BAR proteins and $U_{1,2} \approx -0.9$ nm for the deep immersion state of the H0 helices of N-BAR proteins. For the effective material parameters characterizing lipid bilayer elastic properties, we used the values $K_b \approx 20\ k_BT$, $K_t \approx 60\ k_BT/\mathrm{nm}^2$, and $a \approx 2.0$ nm, which are typical for phospholipid bilayers [9, 60].

In Eq. (S16), the derivatives of the bilayer thickness deformation field along the bilayer-protein interface are specified by $U'_{1,2}$. As noted in Sec. S2, there has been some debate [5, 7, 11–14, 21, 25, 33, 53–58, 61], in the context of integral membrane proteins, as to whether the derivatives of the bilayer thickness deformation field along the bilayer-protein interface should be regarded as fixed, or whether the bilayer-protein interaction energy should be minimized with respect to these derivatives. Previous studies have shown that, with the derivatives of the bilayer thickness deformation field along the bilayer-protein interface set to zero, the classic elastic model of lipid bilayer thickness deformations due to integral membrane proteins can explain the basic experimental phenomenology of the gating

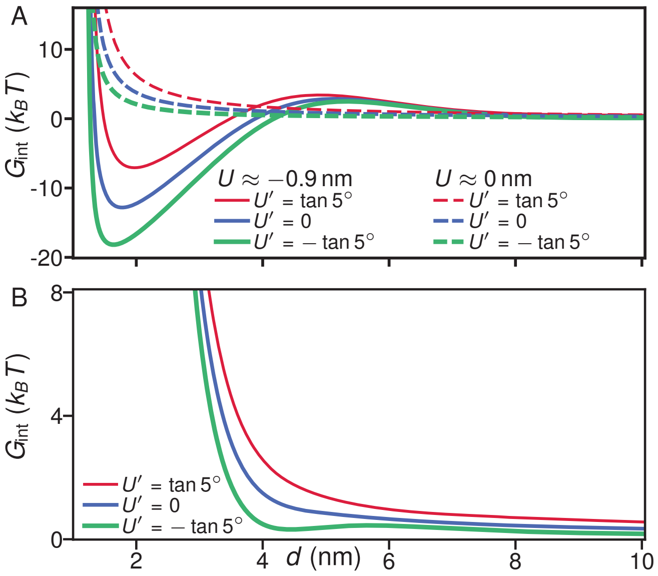

Figure S3: Interaction potential G_{int} between two parallel H0 helices of neighboring N-BAR proteins as in Fig. 3 of the main text but using the indicated values of $U'_1 = U'_2 = U'$ for the measured shallow ($U \approx 0$ nm) and deep ($U \approx -0.9$ nm) membrane immersion states of the H0 helices of N-BAR proteins [1, 2], with (**A**) both H0 helices in the same immersion state and (**B**) one H0 helix in the shallow and one H0 helix in the deep immersion state.

of gramicidin channels [7, 62, 63] and the mechanosensitive channel of large conductance [6, 9, 64, 65]. It has also been found [25] that, if the bilayer-protein interaction energy is optimized with respect to the derivatives of the bilayer thickness deformation field along the bilayer-protein interface, the classic elastic model of lipid bilayer thickness deformations due to integral membrane proteins can produce excellent agreement with the results of MD simulations of protein-induced lipid bilayer deformations. Optimization of the bilayer-protein interaction energy with respect to the derivatives of the bilayer thickness deformation field along the bilayer-protein interface thereby resulted in contact slopes of the bilayer thickness deformation field that tended to be small and positive [25], with some variability along the bilayer-protein interface.

In Fig. S3 we examine the dependence of the bilayer-mediated wedge interactions predicted by our model on $U'_{1,2}$. We thereby set $U'_1 = U'_2 \equiv U'$ and, based on the aforementioned studies of bilayer-protein interactions for integral membrane proteins [6, 7, 9, 62–65], perturb U' about $U' \approx 0$. With the possible exception of lipid bilayers enriched in lipids with substantial intrinsic curvature [66], wedge-induced leaflet thickness deformations are expected to primarily compress or expand the hydrophobic thickness of the lipids surrounding the protein wedge, rather than primarily perturb the hydrophobic thickness of lipids further away from the protein wedge with little compression or expansion of the

hydrophobic thickness of the lipids surrounding the protein wedge. While, for completeness, we allow in Fig. S3 for non-zero U' for the shallow H0 immersion state with $U \approx 0$, we therefore expect that protein wedges with $U \approx 0$ show $U' \approx 0$. We find that, for the parameter values in Fig. S3, the strength of unfavorable bilayer-mediated wedge interactions for the shallow immersion state of the H0 helices [1, 2] tends to increase with increasing U'. Furthermore, Fig. S3 suggests that an increase in U' results in a slight increase in the characteristic separation of two H0 helices in the deep immersion state predicted by our model. We also find that the magnitude of the dimerization energy of the H0 helices in the deep immersion state found in the main text increases with decreasing U'. Finally, we note from Fig. S3 that $U' < 0$ can, in principle, produce a non-monotonic interaction potential also for cases in which at least one H0 helix is in the shallow immersion state with $U \approx 0$, provided that $|U'|$ is large enough. As noted above, however, we generally do not expect that protein wedges with $U \approx 0$ show substantial $|U'|$. We thus find from Fig. S3 that the basic form of the predicted bilayer-mediated interaction potentials between protein wedges is robust with respect to variations in U'. We follow here previous work on the experimental phenomenology of integral membrane proteins [6, 7, 9, 62–65] and set $U'_1 = U'_2 = U' \approx 0$ independent of the wedge immersion depth, while noting that detailed numerical estimates of the magnitude of bilayer-mediated wedge interactions may require a closer examination of the values of $U'_{1,2}$.

Finally, we consider the values of the lipid bilayer leaflet gradients $H'_{1,2}$ in Eq. (S13). A simple estimate of $H'_{1,2}$ can be obtained from the helical wheel of the H0 helices of endophilin [1]. To this end, we define the hydrophobicity angle of an amphipathic helix, α, as one-half of the angle associated with the hydrophobic portion of the helical wheel of the amphipathic helix. The helical wheel of the H0 helices of endophilin suggests that the boundaries between the hydrophobic and hydrophilic portions of the H0 helix roughly lie at residues 7-14 and residues 2-9 or 9-16 in the helical wheel of the H0 helix in Fig. 2B of Ref. [1], yielding a range $\alpha \approx 70°$–$80°$ for α. We expect that it is energetically favorable for $h^+(r)$ to connect to the H0 helix at the boundaries of the hydrophobic portion of the helical wheel so that the interfaces between lipid headgroups and lipid tails match the boundaries of the hydrophobic portion of the α-helix, with the normals of $h^+(r)$ at the lipid-helix interfaces being parallel to the helix radius at that boundaries. On this basis we obtain the estimate $H'_{1,2} = -\tan(90° - \alpha)$, yielding $-\tan 20° \lessapprox H'_{1,2} \lessapprox -\tan 10°$ for the H0 helices of endophilin.

For a more detailed estimate of $H'_{1,2}$ in Eq. (S13) we draw on a previously-developed approach for describing wedge-induced lipid bilayer deformations [34, 35, 46, 48], which is complementary to our minimal elastic model of bilayer-mediated wedge interactions. In this modeling approach, lipid bilayers are described as three-dimensional continuous elastic materials, which deform as rigid, rodlike

inclusions representing protein helices are inserted into their interior. Importantly, this modeling approach yields estimates of the membrane curvature induced by rodlike inclusions as a function of helix immersion depth [46], which, as described below, we can use to estimate $H'_{1,2}$ in Eq. (S13).

In particular, we can estimate $H'_{1,2}$ in Eq. (S13) based on Fig. 7 of Ref. [46], which provides a comprehensive set of estimates of the intrinsic (spontaneous) curvature induced by a rodlike inclusion as a function of the position of the center of the inclusion above the bilayer midplane, $z_{\rm inc}$, for lipid bilayer leaflets that are laterally coupled (no lipid exchange) as well as laterally uncoupled (lipid exchange permitted). Quantitative studies of membrane remodeling are typically based on *in vitro* assays such as in Refs. [1,2,41,67–71], for which the scenario of laterally coupled lipid bilayer leaflets is thought to be most relevant [46]. We therefore focus here on the results in Fig. 7 of Ref. [46] pertaining to laterally coupled lipid bilayer leaflets. The computations yielding Fig. 7 of Ref. [46] were performed for a lipid bilayer with a total thickness of each unperturbed lipid bilayer leaflet equal to 2 nm, with one third of the thickness corresponding to the lipid headgroups and the remaining two thirds corresponding to the lipid tails, yielding an unperturbed hydrophobic tail length equal to 4/3 nm. To translate the results in Fig. 7 of Ref. [46] to the unperturbed lipid tail length $a = 2$ nm considered here we calculate the insertion ratio $\mathcal{R} \equiv |U|/a$. As mentioned above, EPR experiments [1,2] suggest $U \approx 0$ nm for the shallow immersion state of the H0 helices of N-BAR proteins and $U \approx -0.9$ nm for the deep immersion state of the H0 helices of N-BAR proteins. As a result, we obtain $\mathcal{R} \approx 0\%$ for the shallow H0 immersion state and $\mathcal{R} \approx 45\%$ for the deep H0 immersion state, translating to $U \approx 0$ and $|U| \approx 0.6$ nm for the shallow and deep H0 immersion states for the lipid bilayer thickness considered in Fig. 7 of Ref. [46]. We thus obtain $z_{\rm inc} \approx 1.3$ nm and $z_{\rm inc} \approx 0.73$ nm for the shallow and deep H0 immersion states. Using these estimates of $z_{\rm inc}$, together with the computational results in Fig. 7 of Ref. [46] for laterally coupled lipid bilayer leaflets, we estimate that the H0 helices in the shallow immersion state yield an intrinsic curvature $c_0 \approx 0.33$ nm^{-1}, while the H0 helices in the deep immersion state yield $c_0 \approx 0.07$ nm^{-1}. A wedge-induced intrinsic curvature c_0 can be viewed as a circular arc curving the upper lipid bilayer leaflet at the interface between lipid heads and lipid tails, with radius of curvature $r_c = 1/c_0$ and an approximate projected width $2r_0$ along the r-axis (Fig. S1). We hence obtain the estimate $H'_{1,2} = -\tan(\sin^{-1}(r_0 c_0))$, suggesting that $H'_{1,2} \approx -\tan 11^\circ$ for the shallow H0 immersion state and $H'_{1,2} \approx -\tan 2.4^\circ$ for the deep H0 immersion state.

In summary, the above two approaches for estimating $H'_{1,2}$, based on the hydrophobicity angle of the H0 helices of endophilin [1] and a three-dimensional elastic model of protein wedging [46], suggest that $-\tan 20^\circ \lessapprox H'_{1,2} \lessapprox -\tan 10^\circ$ and $-\tan 11^\circ \lessapprox H'_{1,2} \lessapprox -\tan 2^\circ$, respectively. A crucial difference between these two approaches for estimating $H'_{1,2}$ is that the former approach implies a

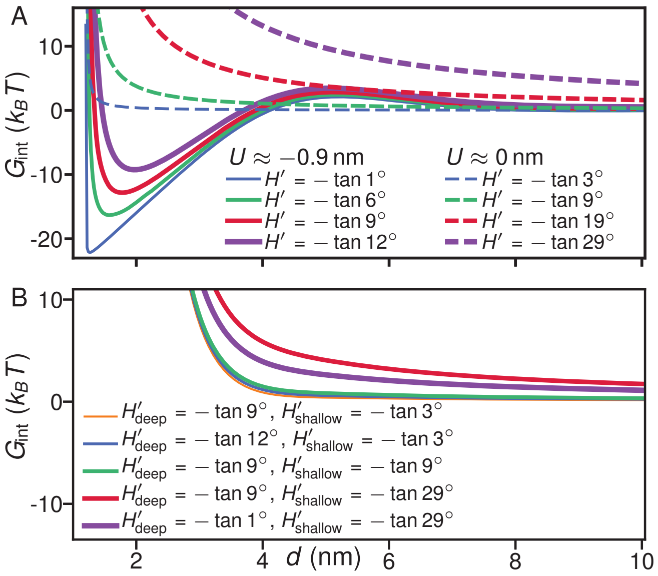

Figure S4: Interaction potential $G_{\rm int}$ between two parallel H0 helices of neighboring N-BAR proteins as in Fig. 3 of the main text but using the indicated values of H'_1 and H'_2 for the measured shallow ($U \approx 0$ nm) and deep ($U \approx -0.9$ nm) membrane immersion states of the H0 helices of N-BAR proteins [1, 2], with (**A**) both H0 helices in the same immersion state and (**B**) one H0 helix in the shallow and one H0 helix in the deep immersion state.

constant $H'_{1,2}$, while the latter approach implies that $H'_{1,2}$ changes with wedge immersion depth. In particular, shallow wedge immersion depths are expected to yield increased values of $|H'_{1,2}|$ [46]. The maximum intrinsic curvature in Fig. 7 of Ref. [46] is given by $c_0 \approx 0.8$ nm^{-1}, which corresponds to $H'_{1,2} \approx -\tan 29°$ in our model. To explore the effect of the value of $H'_{1,2}$ on bilayer-mediated wedge interactions, we recalculated the energy potentials for lipid bilayer-mediated wedge interactions predicted by our model for a range of different values of $H'_{1,2}$ (see Fig. S4). In particular, we considered $-\tan 12° \leq H'_{1,2} \leq -\tan 1°$ for H0 helices in the deep immersion state, and $-\tan 29° \leq H'_{1,2} \leq -\tan 3°$ for H0 helices in the shallow immersion state. For all scenarios considered, we find that the main effect of increasing $|H'_{1,2}|$ is to yield a more pronounced repulsive component of the bilayer-mediated wedge interactions predicted by our model. However, we also find that, similarly as for U' in Fig. S3, the basic form of the predicted bilayer-mediated interaction potentials between protein wedges is robust with respect to variations in $H'_{1,2}$. For the sake of simplicity in our model assumptions, we therefore chose a value of $H'_{1,2}$ intermediate between the approximate minimum and maximum values $H'_{1,2} \approx -\tan 15°$ and $H'_{1,2} \approx -\tan 2°$ suggested by the above estimates, and used a constant $H'_1 = H'_2 \equiv H' \approx -\tan 9°$ independent of the wedge immersion depth. As for U', we note that detailed numerical estimates of the magnitude of bilayer-mediated wedge interactions may require a

Table S1: Dependence of U_c on selected model parameters. In the main text we used $K_b \approx 20\ k_BT$, $a \approx 2.0$ nm, $U' \approx 0$, and $H' \approx -\tan 9^\circ$. For all scenarios we set the lipid bilayer thickness deformation modulus $K_t \approx 60\ k_BT/\mathrm{nm}^2$ and calculated bilayer-mediated wedge interactions as in Fig. 3 of the main text.

K_b	a	U'	H'	U_c
$20\ k_BT$	2.0 nm	0	$-\tan 9^\circ$	-0.40 nm
$14\ k_BT$	1.6 nm	0	$-\tan 9^\circ$	-0.33 nm
$30\ k_BT$	1.6 nm	0	$-\tan 9^\circ$	-0.40 nm
$14\ k_BT$	2.2 nm	0	$-\tan 9^\circ$	-0.39 nm
$30\ k_BT$	2.2 nm	0	$-\tan 9^\circ$	-0.47 nm
$20\ k_BT$	2.0 nm	$\tan 5^\circ$	$-\tan 9^\circ$	-0.61 nm
$20\ k_BT$	2.0 nm	$-\tan 5^\circ$	$-\tan 9^\circ$	-0.21 nm
$20\ k_BT$	2.0 nm	0	$-\tan 6^\circ$	-0.27 nm
$20\ k_BT$	2.0 nm	0	$-\tan 12^\circ$	-0.54 nm

closer examination of the values of $H'_{1,2}$.

In the main text we find that there is a critical membrane immersion depth of the H0 helices, U_c, governing the crossover from unfavorable to favorable bilayer-mediated interactions between two H0 helices in the same membrane-bound conformational state. In particular, we obtain unfavorable bilayer-mediated H0 interactions for $|U| < |U_c|$, but find that bilayer-mediated H0 interactions can become favorable for $|U| > |U_c|$, where $U_c = -0.4$ nm in Fig. 3 of the main text. The numerical value of U_c depends, on the one hand, on the elastic properties of the lipid bilayer, which can be changed by changing the lipid composition. In Table S1 we explore how U_c varies as the lipid bilayer bending rigidity, K_b, and the unperturbed thickness of the monolayer hydrophobic core, a, are varied over the approximate ranges of these elastic parameters measured for phospholipid bilayers [60]. Furthermore, we also explore in Table S1 how U_c changes as U' and H' are varied. Table S1 illustrates that the numerical value of U_c depends on the particular parameter values considered in the model, but that the predicted transition from unfavorable to favorable bilayer-mediated H0 interactions with increasing H0 membrane immersion depth is robust with respect to variations in these model parameters.

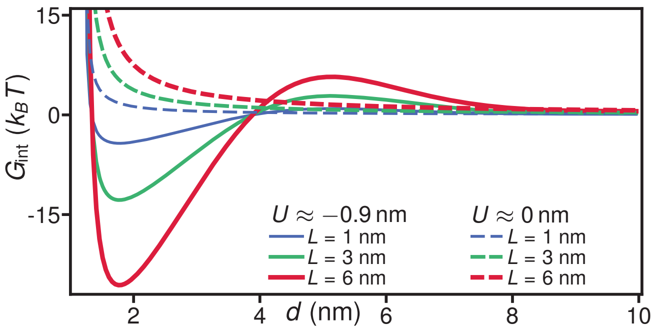

Figure S5: Interaction potential G_{int} between two parallel H0 helices of neighboring N-BAR proteins as in Fig. 3 of the main text but using the indicated values of the wedge length L for the measured shallow ($U \approx 0$ nm) and deep ($U \approx -0.9$ nm) immersion states of the H0 helices of N-BAR proteins [1, 2], with both H0 helices in the same immersion state.

S4 Strength of bilayer-mediated wedge interactions increases with wedge length

The strength of bilayer-mediated wedge interactions is expected to increase with increasing wedge length L. Within the approximation of an effectively one-dimensional system employed for Figs. 3, 5, and 6 of the main text, the magnitude of the interaction energy scales linearly with the wedge length (see Fig. S5). Consequently, for H0 helices in the shallow immersion state [1,2], bilayer-mediated wedge interactions become increasingly unfavorable with increasing helix length. Similarly, the strength of favorable interactions between H0 helices in the deep immersion state [1, 2] increases with increasing wedge length, yielding an increase in the magnitude of the dimerization energy of two H0 helices in the deep immersion state. It is important to note that, as the wedge length is decreased, finite-size effects due to the wedge tips are expected to become increasingly relevant, making it necessary to employ a two-dimensional modeling approach (as, e.g., in Fig. 4 of the main text) rather than the one-dimensional modeling approach used for Fig. S5 (as well as, e.g., Figs. 3, 5, and 6 of the main text).

S5 Bilayer-mediated wedge interactions in curved membrane geometries

As noted in the main text, we generally follow here previous theoretical and computational studies of protein-induced bilayer deformations [5,7,9,21,23,24,33,39,40,72,73] and assume that the unperturbed reference state of the lipid bilayer corresponds to a flat surface with $h^{\pm} = u^{\pm} = 0$. For completeness, we consider in this section the analytic solution of bilayer-mediated wedge interactions in Sec. S2 for a system in which the unperturbed reference state of the lipid bilayer is not flat but shows a radius of curvature $R \approx 15$–25 nm [50, 70, 71, 74]. The corresponding bilayer-mediated wedge interactions are plotted in Fig. S6. As in Sec. S2, we obtained G_{int} in Fig. S6 from G in Eq. (S21) by subtracting G_0

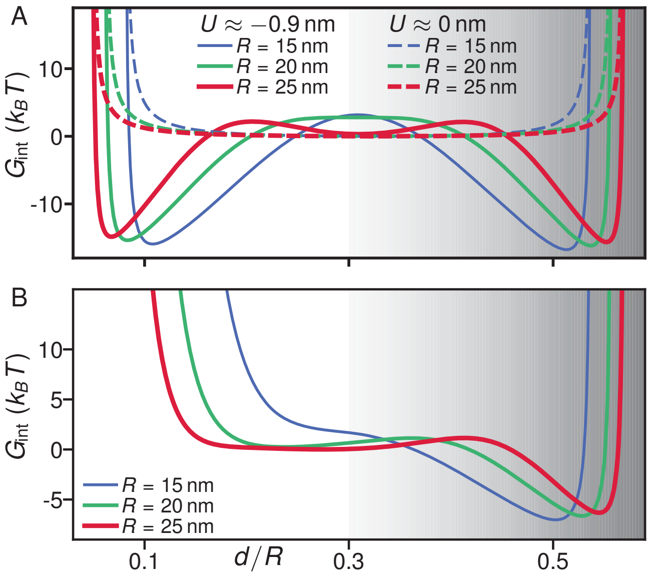

Figure S6: Interaction potential G_{int} between two parallel H0 helices of neighboring N-BAR proteins as in Fig. 3 of the main text but for curved membrane geometries with the indicated radii of curvature R for the measured shallow ($U \approx 0$ nm) and deep ($U \approx -0.9$ nm) immersion states of the H0 helices of N-BAR proteins [1,2], with (**A**) both H0 helices in the same immersion state and (**B**) one H0 helix in the shallow and one H0 helix in the deep immersion state. The gray areas indicate the approximate d-range for which the H0 helices predominantly interact with the fixed boundary conditions imposed by the system geometry, rather than with each other.

in Eq. (S22), which corresponds to the energy of wedge-induced bilayer deformations for $R \to \infty$ and $d \to \infty$.

For the calculations in Fig. S6 we proceeded similarly as in Sec. S2 and assumed that interactions between a given H0 helix and the remainder of the N-BAR protein force the line symmetrically bisecting the hydrophilic and hydrophobic portions of the H0 cross-section to point towards the center of curvature of the undeformed reference state (with now R taking a finite value rather than $R \to \infty$ as in Sec. S2). Hence, we did not allow the wedges to tilt in response to bilayer-mediated wedge interactions. We used a projected total system size (along the r-axis in Fig. S1) corresponding to an arc length of the undeformed reference state equal to $2\pi R/10$. For the boundary conditions at the left and right system boundaries we used the values of $h^+(r)$ and its derivative mandated by a circle of radius R, with both $u^+(r)$ and its derivative being equal to zero. We obtained the heights $H_{1,2}$ of the two wedges relative to the center of curvature of the undeformed reference state by applying zero-force boundary conditions analogous to Eq. (S14) at the wedge boundaries facing towards the system boundaries, and fixed all other boundary conditions as described in Secs. S2 and S3. We also

considered the case in which a zero-force boundary condition as in Eq. (S14) was applied for one of the wedges, with a zero-force boundary condition analogous to Eq. (S14) on the other wedge at the wedge boundary facing towards the system boundary. This latter set of boundary conditions yielded similar results as in Fig. S6. In the small-d regime with substantial bilayer-mediated wedge interactions, indicated in white in Fig. S6, we find bilayer-mediated wedge interactions similar to those in Fig. 3 of the main text. At large d, the H0 helices in Fig. S6 no longer substantially interact with each other but, instead, interact strongly with the fixed boundary conditions imposed by the system geometry. This, possibly unphysical, regime is indicated in gray in Fig. S6.

S6 Simulated annealing Monte Carlo simulations

To test whether lipid bilayer-thickness-mediated interactions between N-BAR proteins due to H0-induced leaflet thickness deformations can yield self-assembly of extended tip-to-tail N-BAR chains with the observed antiparallel alignment and dimerization of neighboring H0 helices [41, 67–71] we calculated the directional N-BAR pair potentials due to H0-induced leaflet thickness deformations in two-dimensional membranes (see Fig. S7A). We computed these bilayer-thickness-mediated interaction potentials $G_{\text{int}}(d, \omega_1, \omega_2)$, where d is the center-to-center distance between the two wedges and the $\omega_{1,2}$ capture the wedge orientations (Fig. S7A), by minimizing the energy in Eq. (3) of the main text for $h^+ = 0$ using finite elements (FE) as described in Ref. [21]. To avoid numerical issues arising from sharp corners of the integration domain we approximated, when computing $G_{\text{int}}(d, \omega_1, \omega_2)$, the shape of the H0 helices of N-BAR proteins as rounded rectangles of total length $L = 3$ nm along the helix axis, with the helix tips modeled as semicircles of radius $r_0 = 0.6$ nm (Fig. S7A). Along the straight long sides of the rounded rectangles we imposed a constant hydrophobic mismatch $U \approx -0.9$ nm corresponding to the deep immersion state of the H0 helices of N-BAR proteins [1, 2], and linearly let $U \to 0$ along the rounded wedge tips towards the centers of the wedge tips. To account for steric constraints on lipid size, we only allowed for wedge separations and orientations with sufficient space for at least one lipid at the closest wedge separation. We implemented this condition by imposing an effective steric constraint on wedge configurations corresponding to a rectangle of length 4 nm and width 2.2 nm centered at the wedge center. Consistent with lipid bilayer-thickness-mediated interactions between integral membrane proteins [75], we find that the bilayer-thickness-mediated interactions between protein wedges in Fig. S7A are approximately pairwise additive (see Fig. S8). We modeled the BAR domains, with one BAR domain connecting two H0 helices (perpendicular to the long axis of the BAR domain) for each N-BAR protein (see Fig. 1 of the main text), as rectangles of length 13 nm and width 2.5 nm with hardcore steric repulsion, with a distance ≈ 4.8 nm between

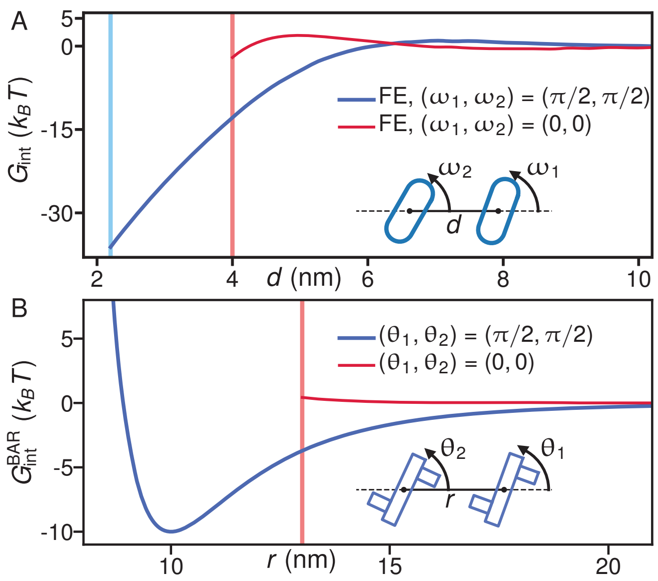

Figure S7: Examples of the directional pair interaction potentials used for Fig. 4 of the main text. (**A**) Lipid bilayer-mediated interactions between N-BAR proteins due to H0-induced leaflet thickness deformations obtained by minimizing Eq. (3) of the main text for $h^+ = 0$ using FE as described in Ref. [21]. (**B**) Lipid bilayer-mediated pair interactions between BAR domains in Eq. (S23), which we modeled after Ref. [39]. The vertical lines indicate steric constraints.

the centers of the BAR domains and the centers of the H0 helices (see Fig. S7B). Based on Ref. [41], we thus approximated the effective, fixed shape of N-BAR proteins setting the hardcore steric constraints on N-BAR configurations as indicated in Fig. 4 of the main text and Fig. S7B.

Lipid bilayer-mediated interactions between BAR domains are expected to favor a face-on orientation of neighboring BAR domains [38–40], with a preferred separation ≈ 10 nm for the endophilin BAR domains [39]. To incorporate bilayer-mediated BAR interactions in Fig. 4B of the main text we employed, based on previous calculations of bilayer-mediated interactions between endophilin BAR domains [39], the modified Lennard-Jones pair potential

$$G_{\text{int}}^{\text{BAR}}(r, \theta_1, \theta_2) = \epsilon \left[\left(\frac{r_m}{r} \right)^{12} - 2 \left(\frac{r_m}{r} \right)^6 \left(1 - \frac{|2\theta_1 - \pi|}{2\pi} - \frac{|2\theta_2 - \pi|}{2\pi} \right) \right] , \tag{S23}$$

where the $\theta_{1,2}$ capture the BAR orientations with a positive modulus π rad and r is the center-to-center distance between BAR domains (Fig. S7B). Based on Ref. [39] we used $\epsilon = 10\ k_BT$ and $r_m = 10$ nm. We obtained the pair interactions between N-BAR proteins used for Fig. 4A of the main text from the pair interactions due to the H0-induced leaflet thickness deformations, and the pair interactions between N-BAR proteins used for Fig. 4B of the main text by adding up the pair interactions due to the BAR domains and the H0-induced leaflet thickness deformations.

For our simulated annealing Monte Carlo simulations of N-BAR pair potentials we proceeded

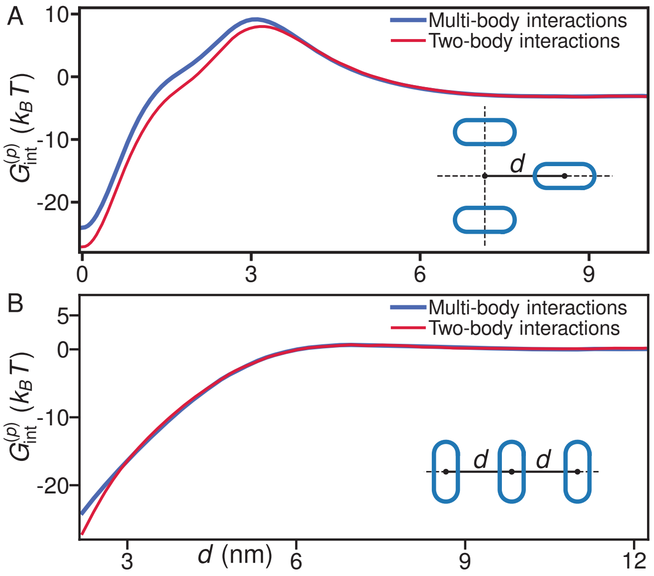

Figure S8: Bilayer-thickness-mediated wedge interactions per protein wedge, $G_{\text{int}}^{(p)}$, for the two wedge configurations indicated in the insets of (**A**) and (**B**) computed, as in Fig. S7A, using FE [21] for the full three-body systems in (A) and (B), and by adding up the corresponding pairwise (two-body) wedge interactions. In panel (A), we use a fixed center-to-center distance between the top and bottom wedges equal to 4.4 nm.

similarly as in Ref. [75]. A single Monte Carlo step in our Monte Carlo simulations consisted, on average, of one displacement trial ($\delta r = 0.1$ nm) and one rotation trial ($\delta\theta = 1°$) per N-BAR protein. The trials were accepted or rejected according to the Metropolis algorithm. In a typical run, we first used 10^7 Monte Carlo steps at constant $T = 2T_{\text{rm}}$, where $T_{\text{rm}} = 298$ K is the room temperature, and then decreased the temperature linearly to $T = 0$ over 5×10^6 Monte Carlo steps. To increase the computational efficiency of our Monte Carlo simulations, we built a table of bilayer-mediated wedge interaction energies with a translational resolution of $\Delta d = 0.25$ nm and an orientational resolution of $\Delta\omega_{1,2} = 3°$. For arbitrary values of (d, ω_1, ω_2), we first rounded ω_1 and ω_2 to their closest values in the table, and then linearly interpolated the bilayer-mediated wedge interaction energy between the two closest values of d in the table.

S7 Concerted structural reorganization of N-BAR proteins

The position-averaged Boltzmann weight associated with a state (s_1, s_2) of two N-BAR proteins in Eq. (6) of the main text [76] is obtained from

$$z(s_1, s_2) = \frac{1}{A^2} \int_A d\mathbf{x}_1 d\mathbf{x}_2 e^{-G_{\text{pair}}(s_1, s_2, d)}, \qquad \text{(S24)}$$

where, following Eq. (4) of the main text,

$$G_{\text{pair}}(s_1, s_2, d) = G_{\text{non}}(s_1, s_2) + G_{\text{int}}(s_1, s_2, d)\,, \tag{S25}$$

the integrals in Eq. (S24) run over the average membrane area A associated with the two N-BAR proteins with the two-dimensional coordinates $\mathbf{x}_{1,2}$ pertaining to the positions of the two N-BAR proteins, and we measure all energies in units of k_BT. Since G_{pair} in Eq. (S24) only depends on the relative position of the two N-BAR proteins, we can rewrite Eq. (S24) as

$$z(s_1, s_2) = \frac{2\pi}{A} e^{-G_{\text{non}}} \int_0^{\sqrt{A/\pi}} \mathrm{d}r e^{-G_{\text{int}}(s_1, s_2, r)} r\,, \tag{S26}$$

where we have assumed a circular patch shape for the H0-induced N-BAR interactions. Defining the Mayer-f function as in Eq. (7) of the main text,

$$f_{12}(s_1, s_2, r) \equiv e^{-G_{\text{int}}(s_1, s_2, r)} - 1\,, \tag{S27}$$

Eq. (S26) becomes

$$z(s_1, s_2) = e^{-G_{\text{non}}} \left[1 + \frac{2\pi}{A} \int_{2r_0}^{\sqrt{A/\pi}} \mathrm{d}r f_{12}(s_1, s_2, r) r \right]\,, \tag{S28}$$

where we have set, for $r < 2r_0$, the contribution to $z(s_1, s_2)$ due to bilayer-mediated wedge interactions equal to zero and, for $r \geq 2r_0$, we take G_{int} to be set by bilayer-mediated wedge interactions as described in the main text. Following previous work [76], we replaced in Eq. (6) of the main text the upper limit of the integral in Eq. (S28) by the cutoff r_c on $G_{\text{int}}(s_1, s_2, r)$, so as to sample the relative N-BAR positions over all values of r yielding a substantial interaction energy between the H0 helices (see Fig. 3 of the main text). As shown in Fig. S9, Eq. (S28) and Eq. (6) of the main text yield similar results for the probability of a pair of N-BAR proteins to be collectively in the observed conformational state with deep immersion of the H0 helices [1, 2], plotted in Fig. 6 of the main text [see Eq. (8) of the main text]. We also show in Fig. S9 the P_d obtained as in Fig. 6 of the main text but using a lower steric cutoff equal to $2r_0 + 1$ nm, rather than $2r_0$, in the integral in Eq. (6) of the main text, thus ensuring that at least one lipid can fit between the wedges for all the wedge configurations considered. This change in the lower cutoff in the integral in Eq. (6) of the main text shifts the curve $P_d = 1/2$, but leaves the key results in Fig. 6 of the main text unchanged.

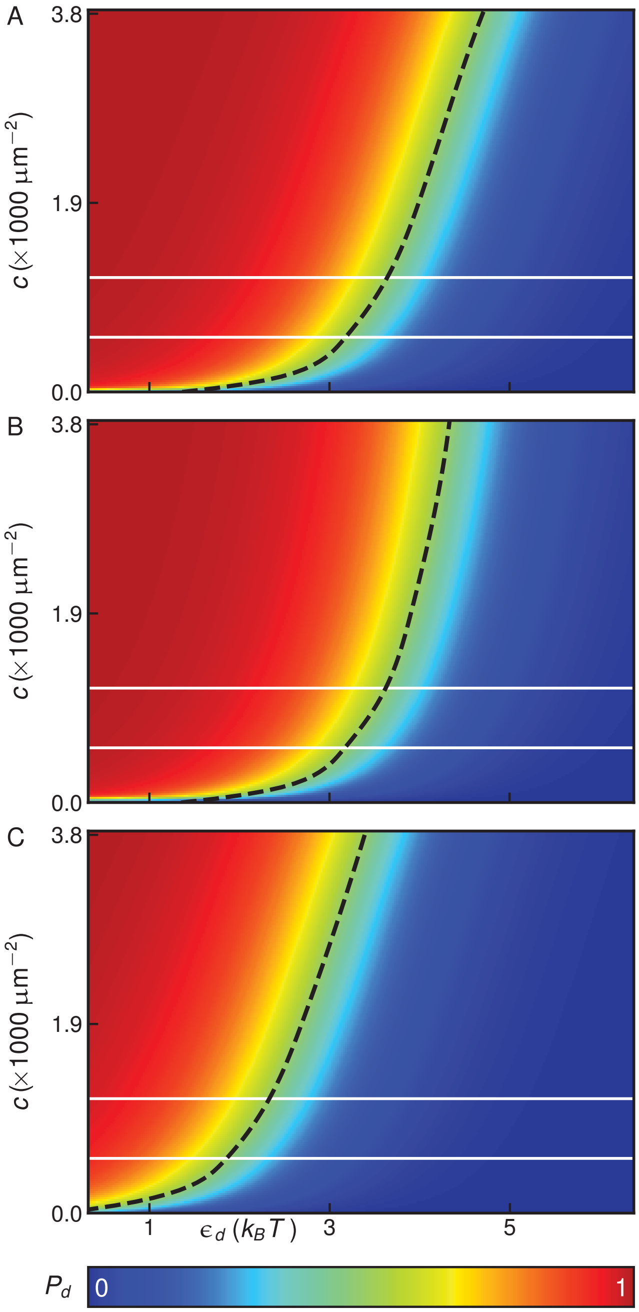

Figure S9: Probability of a pair of N-BAR proteins to be collectively in the observed conformational state with deep immersion of the H0 helices [1,2], P_d, obtained from Eq. (8) of the main text following similar steps as in Fig. 6 of the main text using (**A**) Eq. (6) of the main text, (**B**) Eq. (S28), and (**C**) Eq. (6) of the main text with a lower cutoff in the integral equal to $2r_0 + 1$ nm rather than $2r_0$. In all panels, P_d is plotted over an identical range of ϵ_d and c as in Fig. 6 of the main text, and we use the same labeling conventions as in Fig. 6 of the main text. We use the same color bar for all panels. The data in panel (A) is identical to the data plotted in Fig. 6 of the main text and reproduced here for completeness.

A Variation of the energy of wedge-induced lipid bilayer deformations

For a one-dimensional system, the energy in Eq. (3) of the main text reduces to (see Sec. S2)

$$\begin{aligned} G = \frac{1}{2}\int_{r_1}^{r_2} \mathrm{d}r \Big\{ K_b \Big[\left(\frac{\mathrm{d}^2 h^+}{\mathrm{d}r^2}\right)^2 - \left(\frac{\mathrm{d}^2 h^+}{\mathrm{d}r^2}\right)\left(\frac{\mathrm{d}^2 u^+}{\mathrm{d}r^2}\right) + \frac{1}{2}\left(\frac{\mathrm{d}^2 u^+}{\mathrm{d}r^2}\right)^2 \Big] \\ + \frac{K_t}{2}\left(\frac{u^+}{a}\right)^2 + \tau \frac{u^+}{a} + \tau \Big[\left(\frac{\mathrm{d}h^+}{\mathrm{d}r}\right)^2 - \left(\frac{\mathrm{d}h^+}{\mathrm{d}r}\right)\left(\frac{\mathrm{d}u^+}{\mathrm{d}r}\right) + \frac{1}{2}\left(\frac{\mathrm{d}u^+}{\mathrm{d}r}\right)^2 \Big] \Big\} \\ \equiv \int \mathrm{d}r f(u^+, u_r^+, u_{rr}^+, h_r^+, h_{rr}^+) \,, \end{aligned} \tag{S29}$$

where $r_{1,2} = d/2 \pm r_0$ and we have implicitly defined the energy density f. To derive the equilibrium conditions for Eq. (S29), we consider [24] variations of u^+ and h^+ about the equilibrium solutions u_0 and h_0:

$$u^+(r) = u_0(r) + \epsilon_u \delta u(r) \,, \tag{S30}$$

$$h^+(r) = h_0(r) + \epsilon_h \delta h(r) \,. \tag{S31}$$

For the variations in u^+, we have

$$\frac{\mathrm{d}f}{\mathrm{d}\epsilon_u} = \frac{\partial f}{\partial u^+}\frac{\mathrm{d}u^+}{\mathrm{d}\epsilon_u} + \frac{\partial f}{\partial u_r^+}\frac{\mathrm{d}u_r^+}{\mathrm{d}\epsilon_u} + \frac{\partial f}{\partial u_{rr}^+}\frac{\mathrm{d}u_{rr}^+}{\mathrm{d}\epsilon_u} \,. \tag{S32}$$

Using the product rule, the second and the third terms on the right-hand side of Eq. (S32) can be expressed as

$$\frac{\partial f}{\partial u_r^+}\frac{\mathrm{d}u_r^+}{\mathrm{d}\epsilon_u} = \frac{\partial}{\partial r}\left(\frac{\partial f}{\partial u_r^+}\frac{\mathrm{d}u^+}{\mathrm{d}\epsilon_u}\right) - \frac{\partial}{\partial r}\left(\frac{\partial f}{\partial u_r^+}\right)\frac{\mathrm{d}u^+}{\mathrm{d}\epsilon_u} \tag{S33}$$

and

$$\frac{\partial f}{\partial u_{rr}^+}\frac{\mathrm{d}u_{rr}^+}{\mathrm{d}\epsilon_u} = \frac{\partial}{\partial r}\left(\frac{\partial f}{\partial u_{rr}^+}\frac{\mathrm{d}u_r^+}{\mathrm{d}\epsilon_u}\right) - \frac{\partial}{\partial r}\left(\frac{\partial}{\partial r}\frac{\partial f}{\partial u_{rr}^+}\frac{\mathrm{d}u^+}{\mathrm{d}\epsilon_u}\right) + \frac{\partial^2}{\partial r^2}\left(\frac{\partial f}{\partial u_{rr}^+}\right)\frac{\mathrm{d}u^+}{\mathrm{d}\epsilon_u} \,, \tag{S34}$$

respectively. Thus, we obtain

$$\begin{aligned} \frac{\mathrm{d}G}{\mathrm{d}\epsilon_u} = \int_{r_1}^{r_2} \mathrm{d}r \left\{ \frac{\partial f}{\partial u^+}\frac{\mathrm{d}u^+}{\mathrm{d}\epsilon_u} + \frac{\partial}{\partial r}\left(\frac{\partial f}{\partial u_r^+}\frac{\mathrm{d}u^+}{\mathrm{d}\epsilon_u}\right) \right. \\ \left. - \frac{\partial}{\partial r}\left(\frac{\partial f}{\partial u_r^+}\right)\frac{\mathrm{d}u^+}{\mathrm{d}\epsilon_u} + \frac{\partial}{\partial r}\left(\frac{\partial f}{\partial u_{rr}^+}\frac{\mathrm{d}u_r^+}{\mathrm{d}\epsilon_u}\right) - \frac{\partial}{\partial r}\left[\frac{\partial}{\partial r}\left(\frac{\partial f}{\partial u_{rr}^+}\right)\frac{\mathrm{d}u^+}{\mathrm{d}\epsilon_u}\right] + \frac{\partial^2}{\partial r^2}\left(\frac{\partial f}{\partial u_{rr}^+}\right)\frac{\mathrm{d}u^+}{\mathrm{d}\epsilon_u} \right\} . \end{aligned} \tag{S35}$$

At equilibrium, we must have $\frac{\mathrm{d}G}{\mathrm{d}\epsilon_u} = 0$. Upon performing the integrals for the boundary terms in Eq. (S35), we thus find

$$\begin{aligned} \frac{\mathrm{d}G}{\mathrm{d}\epsilon_u} = \int_{r_1}^{r_2} \mathrm{d}r \left(\frac{\partial f}{\partial u^+} - \frac{\partial}{\partial r}\frac{\partial f}{\partial u_r^+} + \frac{\partial^2}{\partial r^2}\frac{\partial f}{\partial u_{rr}^+}\right)\frac{\mathrm{d}u^+}{\mathrm{d}\epsilon_u} \\ + \left[\left(\frac{\partial f}{\partial u_r^+} - \frac{\partial}{\partial r}\frac{\partial f}{\partial u_{rr}^+}\right)\frac{\mathrm{d}u^+}{\mathrm{d}\epsilon_u} + \left(\frac{\partial f}{\partial u_{rr}^+}\right)\frac{\mathrm{d}u_r^+}{\mathrm{d}\epsilon_u}\right]_{r_1}^{r_2} = 0 \end{aligned} \tag{S36}$$

at equilibrium. From the definition of f in Eq. (S29) we find

$$\begin{aligned}\frac{\partial f}{\partial u^+} &= \frac{K_t}{2a^2}u^+ + \frac{\tau}{2a}\,,\\ \frac{\partial f}{\partial u_r^+} &= \frac{\tau}{2}\left(\frac{\partial u^+}{\partial r} - \frac{\partial h^+}{\partial r}\right)\,,\\ \frac{\partial f}{\partial u_{rr}^+} &= \frac{K_b}{2}\left(\frac{\partial^2 u^+}{\partial r^2} - \frac{\partial^2 h^+}{\partial r^2}\right)\,.\end{aligned} \tag{S37}$$

Substitution of Eq. (S37) into the first term of Eq. (S36) yields the Euler-Lagrange equation

$$\frac{K_b}{2}\left(\frac{\partial^4 u^+}{\partial r^4} - \frac{\partial^4 h^+}{\partial r^4}\right) + \frac{K_t}{2a^2}u^+ + \frac{\tau}{2a} - \frac{\tau}{2}\left(\frac{\partial^2 u^+}{\partial r^2} - \frac{\partial^2 h^+}{\partial r^2}\right) = 0 \tag{S38}$$

associated with $u^+(r)$, which is equivalent to the second Euler-Lagrange equation in Eq. (S6). For the boundary term in Eq. (S36), Eq. (S37) yields

$$\left\{\left[\frac{\tau}{2}\left(\frac{\partial u^+}{\partial r} - \frac{\partial h^+}{\partial r}\right) - \frac{K_b}{2}\left(\frac{\partial^3 u^+}{\partial r^3} - \frac{\partial^3 h^+}{\partial r^3}\right)\right]\frac{\mathrm{d}u^+}{\mathrm{d}\epsilon_u} + \left[\frac{K_b}{2}\left(\frac{\partial^2 u^+}{\partial r^2} - \frac{\partial^2 h^+}{\partial r^2}\right)\right]\frac{\mathrm{d}u_r^+}{\mathrm{d}\epsilon_u}\right\}_{r_1}^{r_2} = 0\,, \tag{S39}$$

where the first and second terms yield the zero force and zero torque boundary conditions [24, 77] associated with u^+, respectively. The boundary conditions in Eqs. (S15) and (S16) imply that the variations in u^+ and its derivative must vanish at $r = r_{1,2}$. Thus, the zero force and zero torque boundary conditions in Eq. (S39) are satisfied by solutions respecting Eqs. (S15) and (S16).

For the variations of the energy in Eq. (S29) with respect to h^+ we follow similar steps as for the variations in u^+ above. In particular, for h^+ we have

$$\begin{aligned}\frac{\mathrm{d}G}{\mathrm{d}\epsilon_h} = &\int_{r_1}^{r_2}\mathrm{d}r\left(\frac{\partial f}{\partial h^+} - \frac{\partial}{\partial r}\frac{\partial f}{\partial h_r^+} + \frac{\partial^2}{\partial r^2}\frac{\partial f}{\partial h_{rr}^+}\right)\frac{\mathrm{d}h^+}{\mathrm{d}\epsilon_h}\\ &+ \left[\left(\frac{\partial f}{\partial h_r^+} - \frac{\partial}{\partial r}\frac{\partial f}{\partial h_{rr}^+}\right)\frac{\mathrm{d}h^+}{\mathrm{d}\epsilon_h} + \left(\frac{\partial f}{\partial h_{rr}^+}\right)\frac{\mathrm{d}h_r^+}{\mathrm{d}\epsilon_h}\right]_{r_1}^{r_2} = 0\end{aligned} \tag{S40}$$

at equilibrium. The definition of f in Eq. (S29) implies the relations

$$\begin{aligned}\frac{\partial f}{\partial h^+} &= 0\,,\\ \frac{\partial f}{\partial h_r^+} &= \tau\left(\frac{\partial h^+}{\partial r} - \frac{1}{2}\frac{\partial u^+}{\partial r}\right)\,,\\ \frac{\partial f}{\partial h_{rr}^+} &= K_b\left(\frac{\partial^2 h^+}{\partial r^2} - \frac{1}{2}\frac{\partial^2 u^+}{\partial r^2}\right)\,.\end{aligned} \tag{S41}$$

Substitution of Eq. (S41) into the first term of Eq. (S40) yields the Euler-Lagrange equation

$$K_b\left(\frac{\partial^4 h^+}{\partial r^4} - \frac{1}{2}\frac{\partial^4 u^+}{\partial r^4}\right) - \tau\left(\frac{\partial^2 h^+}{\partial r^2} - \frac{1}{2}\frac{\partial^2 u^+}{\partial r^2}\right) = 0 \tag{S42}$$

associated with $h^+(r)$, which is equivalent to the first Euler-Lagrange equation in Eq. (S6). For the boundary term in Eq. (S40), Eq. (S41) yields the zero force and zero torque boundary conditions [24,77]

associated with h^+:

$$\left\{\left[\tau\left(\frac{\partial h^+}{\partial r}-\frac{1}{2}\frac{\partial u^+}{\partial r}\right)-K_b\left(\frac{\partial^3 h^+}{\partial r^3}-\frac{1}{2}\frac{\partial^3 u^+}{\partial r^3}\right)\right]\frac{\mathrm{d}h^+}{\mathrm{d}\epsilon_h}+\left[K_b\left(\frac{\partial^2 h^+}{\partial r^2}-\frac{1}{2}\frac{\partial^2 u^+}{\partial r^2}\right)\right]\frac{\mathrm{d}h_r^+}{\mathrm{d}\epsilon_h}\right\}_{r_1}^{r_2}=0\,. \tag{S43}$$

The boundary conditions in Eq. (S13) ensure that the variations of the derivative of $h^+(r)$ vanish at $r = r_{1,2}$. Thus, the zero torque boundary conditions in Eq. (S43) are satisfied by solutions respecting Eq. (S13). We impose, without loss of generality, Eq. (S14), which is obtained from the zero force boundary conditions in Eq. (S43), at $r = r_2$ and set $H_1 = 0$, thus ensuring that the zero force boundary conditions in Eq. (S43) are satisfied.

References

[1] M. R. Ambroso, B. G. Hegde, and R. Langen. Endophilin A1 induces different membrane shapes using a conformational switch that is regulated by phosphorylation. *Proc. Natl. Acad. Sci. U.S.A.*, 111(19):6982–6987, 2014.

[2] J. M. Isas, M. R. Ambroso, P. B. Hegde, J. Langen, and R. Langen. Tubulation by amphiphysin requires concentration-dependent switching from wedging to scaffolding. *Structure*, 23(5):873–881, 2015.

[3] H. W Huang, F.-Y. Chen, and M.-T. Lee. Molecular mechanism of peptide-induced pores in membranes. *Phys. Rev. Lett.*, 92(19):198304, 2004.

[4] S. Ludtke, K. He, and H. Huang. Membrane thinning caused by magainin 2. *Biochemistry*, 34(51):16764–16769, 1995.

[5] H. Aranda-Espinoza, A. Berman, N. Dan, P. Pincus, and S. Safran. Interaction between inclusions embedded in membranes. *Biophys. J.*, 71:648–656, 1996.

[6] P. Wiggins and R. Phillips. Membrane-protein interactions in mechanosensitive channels. *Biophys. J.*, 88:880–902, 2005.

[7] H. W. Huang. Deformation free energy of bilayer membrane and its effect on gramicidin channel lifetime. *Biophys. J.*, 50:1061–1070, 1986.

[8] O. S. Andersen and R. E. Koeppe, II. Bilayer thickness and membrane protein function: An energetic perspective. *Annu. Rev. Biophys. Biomol. Struct.*, 36:107–120, 2007.

[9] R. Phillips, T. Ursell, P. Wiggins, and P. Sens. Emerging roles for lipids in shaping membrane-protein function. *Nature*, 459:379–385, 2009.

[10] J.-B. Fournier. Microscopic membrane elasticity and interactions among membrane inclusions: interplay between the shape, dilation, tilt, and tilt-difference modes. *Eur. Phys. J. B*, 11:261–272, 1999.

[11] G. Brannigan and F. L. H. Brown. A consistent model for thermal fluctuations and protein-induced deformations in lipid bilayers. *Biophys. J.*, 90(5):1501–1520, 2006.

[12] G. Brannigan and F. L. H. Brown. Contributions of Gaussian curvature and nonconstant lipid volume to protein deformation of lipid bilayers. *Biophys. J.*, 92(3):864–876, 2007.

[13] B. West, F. L. H. Brown, and F. Schmid. Membrane-protein interactions in a generic coarse-grained model for lipid bilayers. *Biophys. J.*, 96(1):101–115, 2009.

[14] A.-F. Bitbol, D. Constantin, and J.-B. Fournier. Bilayer elasticity at the nanoscale: The need for new terms. *PLoS ONE*, 7(11):e48306, 2012.

[15] M. C. Watson, E. S. Penev, P. M. Welch, and F. L. H. Brown. Thermal fluctuations in shape, thickness, and molecular orientation in lipid bilayers. *J. Chem. Phys.*, 135(24):244701, 2011.

[16] M. C. Watson, A. Morriss-Andrews, P. M. Welch, and F. L. H. Brown. Thermal fluctuations in shape, thickness, and molecular orientation in lipid bilayers. II. Finite surface tensions. *J. Chem. Phys.*, 139(8):084706, 2013.

[17] P. Rangamani, A. Benjamini, A. Agrawal, B. Smit, D. J. Steigmann, and G. Oster. Small scale membrane mechanics. *Biomech. Model. Mechanobiol.*, 13(4):697–711, 2014.

[18] R. L. Goforth, A. K. Chi, D. V. Greathouse, L. L. Providence, R. E. Koeppe, and O. S. Andersen. Hydrophobic coupling of lipid bilayer energetics to channel function. *J. Gen. Physiol.*, 121(5):477–493, 2003.

[19] H.-J. Kaiser, A. Orłowski, T. Róg, T. K. M. Nyholm, W. Chai, T. Feizi, D. Lingwood, I. Vattulainen, and K. Simons. Lateral sorting in model membranes by cholesterol-mediated hydrophobic matching. *Proc. Natl. Acad. Sci. U.S.A.*, 108(40):16628–16633, 2011.

[20] D. Milovanovic, A. Honigmann, S. Koike, F. Göttfert, G. Pähler, M. Junius, S. Müllar, U. Diederichsen, A. Janshoff, H. Grubmüller, et al. Hydrophobic mismatch sorts SNARE proteins into distinct membrane domains. *Nat. Commun.*, 6:5984, 2015.

[21] O. Kahraman, P. D. Koch, W. S. Klug, and C. A. Haselwandter. Bilayer-thickness-mediated interactions between integral membrane proteins. *Phys. Rev. E*, 93(4):042410, 2016.

[22] A. M. Pollard and V. Sourjik. Transmembrane region of bacterial chemoreceptor is capable of promoting protein clustering. *J. Biol. Chem.*, 293(6):2149–2158, 2018.

[23] M. Goulian, R. Bruinsma, and P. Pincus. Long-range forces in heterogeneous fluid membranes. *Europhys. Lett.*, 22(2):145, 1993.

[24] T. R. Weikl, M. M. Kozlov, and W. Helfrich. Interaction of conical membrane inclusions: Effect of lateral tension. *Phys. Rev. E*, 57:6988–6995, 1998.

[25] D. Argudo, N. P. Bethel, F. V. Marcoline, C. W. Wolgemuth, and M. Grabe. New continuum approaches for determining protein-induced membrane deformations. *Biophys. J.*, 112(10):2159–2172, 2017.

[26] A. R. Braun, E. Sevcsik, P. Chin, E. Rhoades, S. Tristram-Nagle, and J. N. Sachs. α-Synuclein induces both positive mean curvature and negative Gaussian curvature in membranes. *J. Am. Chem. Soc.*, 134(5):2613–2620, 2012.

[27] A. R. Braun, M. M. Lacy, V. C Ducas, E. Rhoades, and J. N. Sachs. α-Synuclein-induced membrane remodeling is driven by binding affinity, partition depth, and interleaflet order asymmetry. *J. Am. Chem. Soc.*, 136(28):9962–9972, 2014.

[28] A. West, B. E. Brummel, A. R. Braun, E. Rhoades, and J. N. Sachs. Membrane remodeling and mechanics: Experiments and simulations of α-synuclein. *Biochim. Biophys. Acta Biomembr.*, 1858(7, Part B):1594 – 1609, 2016.

[29] K. Hristova, W. C. Wimley, V. K. Mishra, G. M. Anantharamiah, J. P. Segrest, and S. H. White. An amphipathic α-helix at a membrane interface: A structural study using a novel X-ray diffraction method. *J. Mol. Biol.*, 290(1):99–117, 1999.

[30] B. S. Perrin, A. J. Sodt, M. L. Cotten, and R. W. Pastor. The curvature induction of surface-bound antimicrobial peptides piscidin 1 and piscidin 3 varies with lipid chain length. *J. Membr. Biol.*, 248(3):455–467, 2015.

[31] S. Y. Woo and H. Lee. Effect of lipid shape on toroidal pore formation and peptide orientation in lipid bilayers. *Phys. Chem. Chem. Phys.*, 19(32):21340–21349, 2017.

[32] S. Y. Woo and H. Lee. Aggregation and insertion of melittin and its analogue MelP5 into lipid bilayers at different concentrations: effects on pore size, bilayer thickness and dynamics. *Phys. Chem. Chem. Phys.*, 19(10):7195–7203, 2017.

[33] N. Dan, A. Berman, P. Pincus, and S. A. Safran. Membrane-induced interactions between inclusions. *J. Phys. II*, 4(10):1713–1725, 1994.

[34] M. M. Kozlov, F. Campelo, N. Liska, L. V. Chernomordik, S. J. Marrink, and H. T. McMahon. Mechanisms shaping cell membranes. *Curr. Opin. Cell Biol.*, 29:53–60, 2014.

[35] F. Campelo, G. Fabrikant, H. T. McMahon, and M. M. Kozlov. Modeling membrane shaping by proteins: Focus on EHD2 and N-BAR domains. *FEBS Lett.*, 584(9):1830–1839, 2010.

[36] T. Baumgart, B. R. Capraro, C. Zhu, and S. L. Das. Thermodynamics and mechanics of membrane curvature generation and sensing by proteins and lipids. *Annu. Rev. Phys. Chem.*, 62:483, 2011.

[37] A. Callan-Jones and P. Bassereau. Curvature-driven membrane lipid and protein distribution. *Curr. Opin. Solid State Mater. Sci.*, 17(4):143–150, 2013.

[38] T. Shemesh, R. W. Klemm, F. B. Romano, S. Wang, J. Vaughan, X. Zhuang, H. Tukachinsky, M. M. Kozlov, and T. A. Rapoport. A model for the generation and interconversion of ER morphologies. *Proc. Natl. Acad. Sci. U.S.A.*, 111(49):E5243–E5251, 2014.

[39] Y. Schweitzer and M. M. Kozlov. Membrane-mediated interaction between strongly anisotropic protein scaffolds. *PLoS Comput. Biol.*, 11(2):e1004054–e1004054, 2015.

[40] H. Noguchi and J.-B. Fournier. Membrane structure formation induced by two types of banana-shaped proteins. *Soft Matter*, 13(22):4099–4111, 2017.

[41] C. Mim and V. M. Unger. Membrane curvature and its generation by BAR proteins. *Trends Biochem. Sci.*, 37(12):526–533, 2012.

[42] H. T. McMahon and J. L. Gallop. Membrane curvature and mechanisms of dynamic cell membrane remodelling. *Nature*, 438(7068):590–596, 2005.

[43] A. Frost, V. M. Unger, and P. De Camilli. The BAR domain superfamily: Membrane-molding macromolecules. *Cell*, 137(2):191–196, 2009.

[44] G. Drin and B. Antonny. Amphipathic helices and membrane curvature. *FEBS Lett.*, 584(9):1840–1847, 2010.

[45] Y. Rao and V. Haucke. Membrane shaping by the Bin/amphiphysin/Rvs (BAR) domain protein superfamily. *Cell. Mol. Life Sci.*, 68(24):3983–3993, 2011.

[46] F. Campelo, H. T. McMahon, and M. M. Kozlov. The hydrophobic insertion mechanism of membrane curvature generation by proteins. *Biophys. J.*, 95(5):2325–2339, 2008.

[47] A. Zemel, A. Ben-Shaul, and S. May. Modulation of the spontaneous curvature and bending rigidity of lipid membranes by interfacially adsorbed amphipathic peptides. *J. Phys. Chem. B*, 112(23):6988–6996, 2008.

[48] F. Campelo and M. M. Kozlov. Sensing membrane stresses by protein insertions. *PLoS Comput. Biol.*, 10(4):e1003556, 2014.

[49] A. J. Sodt and R. W. Pastor. Molecular modeling of lipid membrane curvature induction by a peptide: More than simply shape. *Biophys. J.*, 106(9):1958–1969, 2014.

[50] B. J. Peter, H. M. Kent, I. G. Mills, Y. Vallis, P. J. G. Butler, P. R. Evans, and H. T. McMahon. BAR domains as sensors of membrane curvature: The amphiphysin BAR structure. *Science*, 303(5657):495–499, 2004.

[51] M. Hamm and M. M. Kozlov. Elastic energy of tilt and bending of fluid membranes. *Euro. Phys. J. E: Soft Matter and Biol. Phys.*, 3(4):323–335, 2000.

[52] M. M. Terzi and M. Deserno. Novel tilt-curvature coupling in lipid membranes. *J. Chem. Phys.*, 147(8):084702, 2017.

[53] P. Helfrich and E. Jakobsson. Calculation of deformation energies and conformations in lipid membranes containing gramicidin channels. *Biophys. J.*, 57(5):1075, 1990.

[54] C. Nielsen, M. Goulian, and O. S. Andersen. Energetics of inclusion-induced bilayer deformations. *Biophys. J.*, 74:1966–1983, 1998.

[55] C. Nielsen and O. S. Andersen. Inclusion-induced bilayer deformations: Effects of monolayer equilibrium curvature. *Biophys. J.*, 79(5):2583–2604, 2000.

[56] M. B. Partenskii and P. C. Jordan. Membrane deformation and the elastic energy of insertion: Perturbation or membrane elastic constants due to peptide insertion. *J. Chem. Phys.*, 117:10768–10776, 2002.

[57] M. B. Partenskii, G. V. Miloshevsky, and P. C. Jordan. Stabilization of ion channels due to membrane-mediated elastic interaction. *J. Chem. Phys.*, 118(22):10306–10311, 2003.

[58] M. B. Partenskii, G. V. Miloshevsky, and P. C. Jordan. Membrane inclusions as coupled harmonic oscillators: Effects due to anisotropic membrane slope relaxation. *J. Chem. Phys.*, 120(15):7183–7193, 2004.

[59] V. Raicu and A. Popescu. *Integrated Molecular and Cellular Biophysics*. Springer, 2008.

[60] W. Rawicz, K. C. Olbrich, T. McIntosh, D. Needham, and E. Evans. Effect of chain length and unsaturation on elasticity of lipid bilayers. *Biophys. J.*, 79(1):328–339, 2000.

[61] A. J. Sodt, A. H. Beaven, O. S. Andersen, W. Im, and R. W. Pastor. Gramicidin A channel formation induces local lipid redistribution II: A 3D continuum elastic model. *Biophys. J.*, 112(6):1198–1213, 2017.

[62] T. A. Harroun, W. T. Heller, T. M. Weiss, L. Yang, and H. W. Huang. Experimental evidence for hydrophobic matching and membrane-mediated interactions in lipid bilayers containing gramicidin. *Biophys. J.*, 76:937, 1999.

[63] T. A. Harroun, W. T. Heller, T. M. Weiss, L. Yang, and H. W. Huang. Theoretical analysis of hydrophobic matching and membrane-mediated interactions in lipid bilayers containing gramicidin. *Biophys. J.*, 76:3176, 1999.

[64] P. Wiggins and R. Phillips. Analytic models for mechanotransduction: Gating a mechanosensitive channel. *Proc. Natl. Acad. Sci. U.S.A.*, 101:4071–4076, 2004.

[65] T. Ursell, J. Kondev, D. Reeves, P. A. Wiggins, and R. Phillips. The role of lipid bilayer mechanics in mechanosensation. In A. Kamkin and I. Kiseleva, editors, *Mechanosensitivity in Cells and Tissues 1: Mechanosensitive Ion Channels*, pages 37–70. Springer Press, New York, 2008.

[66] N. Dan and S. A. Safran. Effect of lipid characteristics on the structure of transmembrane proteins. *Biophys. J.*, 75:1410–1414, 1998.

[67] F. Fernandes, L. M. S. Loura, F. J. Chichón, J. L. Carrascosa, A. Fedorov, and M. Prieto. Role of Helix 0 of the N-BAR domain in membrane curvature generation. *Biophys. J.*, 94(8):3065–3073, 2008.

[68] N. Mizuno, C. C. Jao, R. Langen, and A. C. Steven. Multiple modes of endophilin-mediated conversion of lipid vesicles into coated tubes: Implications for synaptic endocytosis. *J. Biol. Chem.*, 285(30):23351–23358, 2010.

[69] H. Cui, C. Mim, F. X. Vázquez, E. Lyman, V. M. Unger, and G. A. Voth. Understanding the role of amphipathic helices in N-BAR domain driven membrane remodeling. *Biophys. J.*, 104(2):404–411, 2013.

[70] C. Mim, H. Cui, J. A. Gawronski-Salerno, A. Frost, E. Lyman, G. A Voth, and V. M. Unger. Structural basis of membrane bending by the N-BAR protein endophilin. *Cell*, 149(1):137–145, 2012.

[71] B. Daum, A. Auerswald, T. Gruber, G. Hause, J. Balbach, Kühlbrandt W., and A. Meister. Supramolecular organization of the human N-BAR domain in shaping the sarcolemma membrane. *J. Struct. Biol.*, 194(4):375–382, 2016.

[72] M. Simunovic, A. Srivastava, and G. A. Voth. Linear aggregation of proteins on the membrane as a prelude to membrane remodeling. *Proc. Natl. Acad. Sci. U.S.A.*, 110(51):20396–20401, 2013.

[73] M. Simunovic and G. A. Voth. Membrane tension controls the assembly of curvature-generating proteins. *Nat. Commun.*, 6:7219, 2015.

[74] J. L. Gallop, C. C. Jao, H. M. Kent, P. J. G. Butler, P. R. Evans, R. Langen, and H. T. McMahon. Mechanism of endophilin N-BAR domain-mediated membrane curvature. *EMBO J.*, 25(12):2898–2910, 2006.

[75] O. Kahraman, P. D. Koch, W. S. Klug, and C. A. Haselwandter. Architecture and function of mechanosensitive membrane protein lattices. *Sci. Rep.*, 6:19214, 2016.

[76] T. Ursell, K. C. Huang, E. Peterson, and R. Phillips. Cooperative gating and spatial organization of membrane proteins through elastic interactions. *PLoS Comput. Biol.*, 3(5):e81, 2007.

[77] L. D. Landau and E. M. Lifshitz. *Theory of Elasticity.* Pergamon Press, Oxford, UK, 3rd edition, 1986.
